# Strategies towards Improved Feed Efficiency in Pigs Comprise Molecular Shifts in Hepatic Lipid and Carbohydrate Metabolism

**DOI:** 10.3390/ijms18081674

**Published:** 2017-08-01

**Authors:** Henry Reyer, Michael Oster, Elizabeth Magowan, Dirk Dannenberger, Siriluck Ponsuksili, Klaus Wimmers

**Affiliations:** 1 Institute for Genome Biology, Leibniz Institute for Farm Animal Biology (FBN), Wilhelm-Stahl-Allee 2, 18196 Dummerstorf, Germany; reyer@fbn-dummerstorf.de (H.R.); oster@fbn-dummerstorf.de (M.O.); s.wimmers@fbn-dummerstorf.de (S.P.); 2 Agri-Food and Biosciences Institute, Co. Down, Hillsborough BT26 6DR, UK; Elizabeth.Magowan@afbini.gov.uk; 3 Institute for Muscle Biology and Growth, Leibniz Institute for Farm Animal Biology (FBN), Wilhelm-Stahl-Allee 2, 18196 Dummerstorf, Germany; dannenberger@fbn-dummerstorf.de; 4 Faculty of Agricultural and Environmental Sciences, University Rostock, 18059 Rostock, Germany

**Keywords:** feed efficiency, gene expression, lipid metabolism, liver, nutrient utilization, resource efficiency

## Abstract

Due to the central role of liver tissue in partitioning and metabolizing of nutrients, molecular liver-specific alterations are of considerable interest to characterize an efficient conversion and usage of feed in livestock. To deduce tissue-specific and systemic effects on nutrient metabolism and feed efficiency (FE) twenty-four animals with extreme phenotypes regarding residual feed intake (RFI) were analyzed. Transcriptome and fatty acid profiles of liver tissue were complemented with measurements on blood parameters and thyroid hormone levels. Based on 803 differentially-abundant probe sets between low- and high-FE animals, canonical pathways like integrin signaling and lipid and carbohydrate metabolism, were shown to be affected. Molecular alterations of lipid metabolism show a pattern of a reduced hepatic usage of fatty acids in high-FE animals. Complementary analyses at the systemic level exclusively pointed to increased circulating triglycerides which were, however, accompanied by considerably lower concentrations of saturated and polyunsaturated fatty acids in the liver of high-FE pigs. These results are in accordance with altered muscle-to-fat ratios usually ascribed to FE animals. It is concluded that strategies to improve FE might favor a metabolic shift from energy storage towards energy utilization and mobilization.

## 1. Introduction

The efficient usage of resources is a major concern in agri-food production. It has been shown that feed efficiency (FE) of pigs is largely influenced by husbandry environment including dietary composition and dietary energy concentration [1,2]. Moreover, animal-intrinsic factors play an important role in improvement of FE. This is reflected by considerable improvement of FE due to the implementation of genetic information in breeding programs over the last decades [3]. However, recent genetic analyses still revealed moderate heritabilities for most common FE measurements indicating a substantial genetic potential for further improvement [4]. Indeed, the estimated heritability for complex traits indicating FE like feed conversion ratio (FCR; ratio of feed intake and weight gain) and residual feed intake (RFI; used as a metric for FE, which gives the differences between actual and predicted feed intake) ranged between 0.3 and 0.4 in three different pig breeds, analyzed in a recent study [4]. In the same study, the analyses of genetic parameters of FE-related traits showed strong correlations between both FE traits (RFI and FCR) and daily feed intake. Accordingly, FE is dependent on systemic processes, which primarily rely on (i) the regulation of appetite via the hypothalamus and gut hormones, (ii) absorption processes in the intestine, (iii) partitioning and metabolizing of nutrients in the liver, and (iv) accumulating both muscle and adipose tissue to increase gain in body mass. Specifically, the liver plays a prominent and central role regarding the quantitative and qualitative utilization of absorbed nutrients, including storage, de-novo synthesis, and recycling of nutritive components. Macronutrients like carbohydrates and proteins are metabolized in the liver ensuring a homeostatic maintenance of the nutrient ratio in the peripheral blood. Therefore, the liver is able to store nutrients for later release into the blood (e.g., triacylglycerols), but also has synthesis capacities (e.g., ketone bodies). Lipids primarily bypass the porcine liver [5]. Nevertheless, a considerable hepatic influx of lipids is expected to occur due to the high caloric diet of finishing pigs [6,7]. Adaptation to variable nutrient levels is ensured via the interrelated metabolic routes comprising hepatic lipid, carbohydrate, and protein utilization. There is an increasing body of evidence that the genetic selection towards FE in pigs employs an array of molecular mechanisms, which mainly rely on the routes to utilize macronutrients [8,9] and provoke a direct molecular response at the level of the liver [10]. Indeed, characteristics of lipid metabolism such as lipid and fatty acid profiles [11,12], enzyme activities [13], and associated transcript abundances [11,14,15,16] have been reported to be altered in FE-divergent animals. Consistently, previous studies have suggested that FE classification is related to the processing of carbohydrates [14,17]. The consequences of improved FE on the protein metabolism of the pig are ambiguous but need further investigations due to environmental implications [11,18,19].

Complementary analysis at the phenotypic level showed that high-FE pigs tended to have less body and intramuscular fat and higher lean meat percentage [20,21]. However, there are still many knowledge gaps about the underlying host-based molecular mechanisms. Hence, the critical and central role of hepatocytes regarding both nutrient partitioning and health aspects (e.g., detoxification, oxidative stress) has to be further investigated in FE-divergent animals as suggested previously [22]. The goal of this study was to determine whether hepatic phenotypic traits and hepatic expression profiles reflect FE traits in pigs. Hence, the analyses comprise performance traits, fatty acid concentrations, and transcript abundances deduced from liver tissue of RFI-divergent full-sib pigs.

## 2. Results

The current study investigated growth performance, physiological parameters, and hormones, as well as hepatic fatty acid concentration and hepatic transcript abundance, in pigs divergent in their RFI.

### 2.1. Affected Phenotypic Traits Due to Residual Feed Intake (RFI) Classification

Due to FE measurements and subsequent RFI classification, high-FE animals showed significantly (*p* < 0.05) decreased average daily feed intake (ADFI) and decreased FCR when compared to low-FE animals (Table 1). Moreover, high-FE animals exhibited a significantly increased liver weight. Body weight, average daily weight gain (ADG), and backfat measurements were unaltered between the experimental groups.

Regarding the physiological blood parameters, high-FE animals showed significantly increased triglyceride concentrations in serum when compared to low-FE animals. Parameters reflecting liver function and metabolism such as albumin, glucose, gamma-glutamyl transferase (GGT), glutamate pyruvate transaminase (GPT), glutamate oxaloacetate transaminase (GOT), urea, lactate dehydrogenase (LDH), total cholesterol, and total protein, remained unaltered by FE classification. Moreover, the measured serum T3 and T4 levels did not differ significantly between the experimental groups.

The fatty acid contents of liver tissue are shown in Table 2. Decreased pig liver fat contents were observed in the high-FE group compared to the low-FE group (2.36% vs. 2.57%). This is predominantly based on significantly higher single and sum concentrations of saturated fatty acids (SFA; C16:0 and C18:0) and polyunsaturated fatty acids (PUFA; C18:2 *n*-6, C18:3 *n*-6, C20:2 *n*-6, C20:5 *n*-3, and C22:5 *n*-3) in low-FE animals. In particular, both sum concentrations of *n*-3 PUFA and *n*-6 PUFA were altered between the FE-divergent groups. Single and sum concentrations of monounsaturated fatty acids (MUFA) were unaffected in liver tissue.

### 2.2. Hepatic Gene Expression Pattern

The *snowball* microarray covers 47,845 probe sets, which correspond to 25,024 annotated genes. The initial analyses as described above identified 36,473 probe sets (~76%) for further analysis resulting in 15,486 annotated genes used for further analysis. Transcriptional differences were pronounced due to effects mediated by FE classification. Expression profiling in liver tissue revealed 803 transcripts, which showed significantly altered mRNA abundances between the experimental groups (Table S1). Via integrating gene ontology information, the set of transcripts was used to apply a pathway analysis (threshold: *p* < 0.01), which revealed an FE-dependent enrichment of integrin signaling, ephrin A signaling, adipogenesis pathway, and insulin receptor signaling (Table 3). Enriched bio-functions of “carbohydrate metabolism” revealed the uptake, oxidation, and quantity of carbohydrates as altered between FE-divergent pigs (Table 4). Regarding the ‘lipid metabolism’ theme, differentially abundant transcripts were found to be involved in the hepatic steroid metabolism and the altered concentration of fatty acids in liver tissue. Exclusively, the “transport of neutral amino acid” bio-function was significantly enriched in the “amino acid metabolism” theme. Gene annotations for the most significantly FE-associated probe sets pointed to *SQLE*, *FYN*, and *SLC7A9* (Table S1). Moreover, genes involved in cell interaction like *CLDN3*, *ITGA1*, and *ITGA5* and the regulation of carbohydrate metabolism, like *PDK2*, were among the differentially-expressed genes.

### 2.3. Verification of Microarray Results

To verify differences in mRNA abundance of selected transcripts, the results obtained via microarray and quantitative real-time PCR (RT-qPCR) were analyzed (Table 5). Both the similarity of fold changes (FC) and correlations of expression values between the two systems suggest reliable results. Spearman rank correlation coefficients ranged from 0.69 to 0.94. Taken together, the RT-qPCR results indicate reproducibility of both the microarray data and subsequent statistical analyses.

## 3. Discussion

This study clearly shows that full-sib pigs differ considerably in their FE traits as expressed by RFI, ADFI, and FCR values. Similar differences have been reported in selection experiments based on RFI values as well as in extensively phenotyped animal populations [13,14,23].

### 3.1. Fatty Acid Concentrations in Feed Efficiency (FE)-Divergent Pigs

Animals classified as highly feed efficient have proven to be leaner [17,24], which is indicated by increased muscle to fat ratio. This implies alterations of corresponding metabolic processes [8,17]. In this study backfat thickness was unaffected by experimental group at day 140, which is different from previous reports [14,16]. However, considerable alterations of fatty acid concentrations were found in liver tissue. Overall, pigs classified as high-FE exhibited less hepatic fat content than their low-FE counterparts, which was predominantly based on significantly decreased concentrations of long-chain SFA such as C16:0 and C18:0 and PUFA like C18:2*n*-6 and C20:5*n*-3. The observed effects were consistent among these fatty acids with higher levels in low-FE pigs. Odd-chain fatty acids (i.e., C13:0 and C15:0), which were more prominent in the high-FE group, are discussed as biomarkers for dietary food intake and play a yet unclear role in metabolism [25]. Importantly, a part of saturated fatty acids (C16:0, C18:0) and all PUFAs are known to be de novo synthesized, which primarily takes place in adipose and muscle tissues in pigs [5,26]. Thus, results of this study provide evidence for altered usage of SFA and PUFA in the liver. This could be either driven by decreased incorporation of fatty acids in lipid bilayers of hepatocytes or reduced uptake/storage of fatty acids in the liver of high-FE pigs. Interestingly, the serum level of triglycerides was significantly increased in high-FE animals, thus supporting the latter hypotheses of higher mobilization rates of fatty acids. The FE-divergent pigs showed unaffected transcript abundances of genes involved in lipogenesis such as fatty acid synthase (*FASN*), stearoyl-CoA desaturase (*SCD*), ATP citrate lyase (*ACLY*), acetyl-CoA carboxylases (*ACACA*, *ACACB*), sterol regulatory element-binding protein (*SREBP-1c*), and fatty acid elongases (*ELOVL1*, *ELOVL3*, *ELOVL5*, *ELOVL7*). Expression levels of genes, known to be involved in fatty acid transport, like cluster determinant 36 (*CD36*), fatty acid transport proteins, and fatty acid binding proteins, did also not differ significantly between FE-divergent groups. Interestingly, apolipoprotein A2 (*APOA2*), which is known to be a major high-density lipoprotein (HDL) particle was found to be differentially expressed between groups at a nominal significance level (*p* = 0.028). *APOA2* is proposed as a candidate gene affecting fatty acid composition, albeit results were deduced from adipose tissue [27]. Expression of transcripts associated with hepatic lipid metabolism and utilization like *SIRT1*, *NR1H4*, and *SQLE* differed significantly between experimental groups. *SIRT1* is involved in the regulation of lipid metabolism via cross-talk with the LKV1/AMPK signaling pathway [28]. Consequently, the lower abundance of *SIRT1* in high-FE animals might lead to reduced hepatic fatty acid oxidation compared to low-FE pigs, and eventually results in the development of hepatic steatosis under high-fat diets [29]. However, the integration of expression profiles provide no hints for altered inflammatory processes related to FE status at the level of the liver. Both *NR1H4* and *SQLE* are involved in bile acid metabolism. The former gene encodes the bile acid receptor, which mediates the expression of genes contributing to the synthesis and transport of bile acid. The squalene epoxidase encoding *SQLE* is involved in sterol biosynthesis as a precursor for hepatic bile acid production. Therefore, transcriptional alterations regarding lipid metabolism reveal a pattern of reduced hepatic usage of fatty acids in high-FE animals.

### 3.2. Carbohydrate and Protein Metabolism in FE-Divergent Pigs

Serum levels of glucose, amylase, urea, and total protein remained unaffected by FE traits. However, high-FE pigs showed a significantly higher abundance of *PDK2*. In fact, the protein encoded by *PDK2* is known to inhibit the hepatic pyruvate dehydrogenase complex (PDH), which acts as a gatekeeper in carbohydrate metabolism. Hence, transcriptional responsiveness of *PDK2* might target the acetyl-CoA production mediating a key metabolic switch from glucose utilization towards increased fat metabolism. Additionally, hepatic glucose transporter 2 (*SLC2A2*) was decreased in abundance in high-FE animals (*p* = 0.02), albeit not significantly after multiple testing correction. The transcriptional clues are in line with the assumption that high-FE animals favor energy storage as carbohydrates (e.g., glycogen) rather than as lipids [21,30]. Indeed, complementary studies focusing on muscular phenotypes showed that high-FE pigs exhibited increased muscle glycogen content, lowered pH post-mortem, and higher glycolytic potential [9,17]. In the current study, lower transcript abundance of *NR1H4* might account for modulations in carbohydrate/glycogen metabolism in high-FE pigs [31]. Unless the transcript abundance of amino acid transporters (*SLC1A4*, *SLC7A9*) were different between groups, the results of the current study did not show definite clues regarding their consequences for protein metabolism based on differences in FE, as they have been discussed previously [19].

### 3.3. FYN as a Putative Hub Molecule Regulating FE

The hepatic transcriptome analyses indicate a high responsiveness of metabolic routes and signaling pathways related to cell-cell interaction and cellular growth. Indeed, liver cells are prone to improve cellular infrastructure and intercellular communication as a response to metabolic requirements [24], which directly affects growth and development of the organ [32]. In particular, *FYN* plays a prominent role in the biological pathways designated in Table 3 and was identified as one of the transcripts exhibiting the highest responsiveness to FE traits. Correspondingly, transcriptomic analyses of adipose tissue revealed deceased mRNA abundances of *FYN* in highly efficient animals [24]. Based on the functional annotations of *FYN*, it is involved in cell growth, cell adhesion, integrin-mediated signaling, cytoskeletal remodeling, cell motility, and immune response. Moreover, *FYN* was proposed as major factor regulating fatty acid utilization and glucose homeostasis via effects on AMP-activated protein kinase (AMPK) activity [33]. Mouse models lacking functional *FYN* showed a markedly reduced adipose tissue mass [34], producing a phenotype similar to high-FE animals [24,35]. Indeed, the mouse model revealed reduced triglyceride concentrations in liver and peripheral organs [34], which are consistent with results reported in this study. Interestingly, under fasting conditions these mice exhibited a marked increase in fatty acid utilization and showed higher rates of energy expenditure compared to wild-type controls [34]. As such, the observed decrease in the mRNA abundance of *FYN* in high-FE pigs might represent a molecular adaptation to permanent dietary availability of energy sources. Thus, it is conceivable that FE traits represent a metabolic shift from energy storage towards energy utilization and mobilization. Indeed, high-FE sows underwent considerable losses in fat mass and body weight and exhibited an impaired energy balance during lactation [36].

Furthermore, *FYN* has been shown to affect carbohydrate metabolism via increased insulin sensitivity and glucose metabolism, which have been induced under fasting conditions [34]. In this study, the insulin receptor signaling pathway comprising *FYN* and *INSR* was altered between the FE-divergent groups at the transcriptional level. As such, *FYN* might play a central role in mediating the balance in lipid and carbohydrate metabolism. Consequently, previous studies highlighted *FYN* as a putative hub molecule for processes regulating fatty acid metabolism [37].

### 3.4. Implication on Systemic Integrity of FE-Divergent Pigs

Measurements of blood parameters such as transaminases, LDH, and albumin showed that hepatic liver function was unaffected by FE traits. This provides evidence for the integrity of the liver despite improvements in FE under normal husbandry conditions. In this context it has to be noted that high-FE animals might be prone to metabolic disturbances when facing challenges, such as pregnancy or caloric restriction [16,36]. Moreover, elevated levels of triglycerides, as revealed for high-FE animals in this study, are one of the known risk factors for long-term health consequences such as the metabolic syndrome [38,39].

However, the molecular and physiological findings represent insights for putative strategies to increase FE. As such, high-FE animals might favor metabolic routes employing a higher mobilization and lowered storage of nutrients and an increased cellular communication and infrastructure in hepatocytes, adipocytes, and myocytes [16,40]. Highly efficient animals have been reported to exhibit elevated T_3_ levels [16], whereas in our study T_3_ was only numerically increased in high-FE pigs. Indeed, elevated T_3_ levels stimulate both lipogenesis and lipolysis culminating in fat loss [41] which reflects the phenotype of high-FE animals. Hence, the systemic effects mediated by thyroid hormones might induce a metabolic shift characterized by increased lipolysis and gluconeogenesis [41,42,43].

## 4. Materials and Methods

### 4.1. Animals, Feed Conversion Testing and Sampling

The pig trial was conducted under project licenses PPL 2751 (16 August 2013) and PPL 2781 (27 November 2014) obtained from the Department of Health, Social Services, and Public Safety (DHSSPS). The study was in accordance with the Animals (Scientific Procedures) Act 1986 and followed European Union regulations outlining minimum standards for the protection of pigs (91/630/EEC) and concerning the protection of animals kept for farming purposes (98/58/EC).

Eight F1 sows (Large White × Landrace) were artificially inseminated with semen from four Hylean Maxgro sires (Hermitage Genetics, Kilkenny, Ireland) divergent in their breeding values for FCR. The offspring, comprising 87 piglets of both sexes, were penned by litter after weaning at day 28. Pigs were weighed weekly and feed intake was recorded between days 70 and 140 using Feed Intake Recording Equipment (FIRE) feeding systems (Schauer Agrotronic, Prambachkirchen, Austria). Average body weights of pigs at the start (day 70) and end (day 140) of the trial were 27.8 ± 4.8 kg and 93.9 ± 10.9 kg. During the test period, water and a common pelleted finisher diet (Table S2) were provided ad libitum. The health status of pigs was checked twice per day by observation; signs of illness and treatments were documented. Backfat thickness was measured at day 98, day 119, and day 140 via a non-invasive SonoScape device (Model L3 Veterinary Flat Linear Array, SonoScape, Shenzhen, China). The measurements were taken between the 3rd and 4th last rib, i.e., 5 cm and 7 cm from the midline. Individual FE was assessed by RFI values, including phenotypic measurements obtained between day 70 and day 119 of age. Specifically, the residuals were retrieved from a least squares regression model of average daily feed intake and average daily gain including phenotypic information of metabolic live weight, gender, backfat, and muscle-depth, as described previously [44].

Based on RFI values, pigs were classified as either high-FE (low RFI; mean ± SE: −0.200 ± 0.024) or low-FE (high RFI; mean ± SE: +0.207 ± 0.031). At day 140 of life, all animals were weighed and 24 extremes for FE were selected for sampling under postprandial conditions. The selection considered a balanced design for sex (12 males and 12 females) and litter (eight litters sired by four boars) leading to the sampling of most FE-divergent siblings of the same sex. Selected pigs were in good condition regarding health status and vitality. Pigs were slaughtered by CO_2_ stunning followed by exsanguination. Blood samples were collected from trunk blood. Serum was prepared and samples were stored at –80 °C until use. Liver weight was recorded. Tissue samples of the right liver lobe (*Lobus Spigelii*) were dissected. Tissue samples were snap frozen in liquid nitrogen and stored at –80 °C until RNA and lipid extraction.

### 4.2. Physiological Parameters and Hormones in Serum

Serum samples obtained from animals (*n* = 24) at day 140 were used to analyze physiological parameters (albumin, amylase, GGT, glucose, GOT, GPT, lactate dehydrogenase, lipase, total cholesterol, total protein, triglyceride, urea) via commercial assays (Fuji DriChem 4000i, FujiFilm, Minato, Japan). Serum thyroid hormones T_3_ (triiodothyronine) and T_4_ (thyroxine) were determined in duplicate using a commercially available magnetic bead-based quantitative immunoassay (MAGPIX system) according to manufacturer’s protocols (Merck Millipore, Darmstadt, Germany).

### 4.3. Lipid Extraction and Fatty Acid Profiling

After mincing of frozen liver samples (*n* = 24), approximately 250 mg, 8 mL chloroform/methanol (2:1, *v*/*v*) were added. Total lipids were extracted by the use of Ultra Turrax T25 (IKA, Staufen, Germany), homogenizing in three cycles of 15 s each (12,000 rpm at room temperature). The solution contained C19:0 as an internal standard. The detailed sample preparation procedure has been previously described [45]. Briefly, all of the solvents contained 0.005% (*w*/*v*) of t-butylhydroxytoluene to prevent the oxidation of PUFA. The extraction mixtures were stored at 5 °C for 18 h in the dark and subsequently washed with 0.02% CaCl_2_ solution. The organic phase was separated and dried with Na_2_SO_4_ and K_2_CO_3_ (10:1, *w*/*w*), and the solvent was subsequently removed under gentle nitrogen at room temperature. The lipid extracts were redissolved in 300 μL of toluene, and a 25 mg aliquot was used for methyl ester preparation. Total lipids were stored at –18 °C until transmethylation of fatty acids. Next, 2 mL of 0.5 M sodium methoxide in methanol was added to the samples, which were shaken in a 60 °C water bath for 10 min. Subsequently, 1 mL of 14% boron trifluoride (BF_3_) in methanol was added to the mixture, which was then shaken for an additional 10 min at 60 °C. Saturated NaHCO_3_ solution (2 mL) was added, and the fatty acid methyl esters (FAMEs) were extracted three times in 2 mL of *n*-hexane. The *n*-hexane extract were dried with Na_2_SO_4_ and K_2_CO_3_ (10:1, *w*/*w*), and after filtration reduced to dryness using a vacuum centrifuge (2000 rpm, 30 °C, 30 min). The FAMEs were resuspended in 100 µL of *n*-hexane and stored at –18 °C until used for gas chromatography (GC) analysis.

The fatty acid analysis of the liver lipids was performed using capillary GC with a CP-Sil 88 CB column (100 m × 0.25 mm, Chrompack-Varian, Lake Forest, CA, USA) that was installed in a PerkinElmer gas chromatograph CLARUS 680 with a flame ionization detector and split injection (PerkinElmer Instruments, Shelton, CT, USA). The detailed GC conditions were recently described [46]. Briefly, the initial oven temperature was 150 °C, which was held for 5 min. Subsequently, the temperature was increased to 175 °C and then to 200 °C at a rate of 2 °C min^‒1^ and held for 10 min. Finally, the temperature was increased to 225 °C at a rate of 1.5 °C min^‒1^ and held for 25 min. Hydrogen was used as the carrier gas at a flow rate of 1 mL min^‒1^. The split ratio was 1:20, and the injector and detector were set at 260 °C and 280 °C, respectively. The quantification of fatty acids was done by the use of C19:0 as internal standard. For the calibration procedure the reference standard mixture “Sigma FAME” (Sigma-Aldrich, Deisenhofen, Germany), the methyl ester of C18:1*cis*-11, C22:5*n*-3 and C18:2*cis*-9, *trans*-11 (Matreya, State College, PA, USA), C22:4*n*-6 (Sigma-Aldrich, Deisenhofen, Germany) and C18:4*n*-3 (Larodan, Limhamn, Sweden) were used. The five-point calibration of single fatty acids ranged between 16 and 415 mg/mL and was checked after GC analysis of five samples. Fatty acid concentrations are displayed as mg/100 g liver tissue.

### 4.4. RNA Isolation

For expression analyses, a subset of animals was selected aiming for a balanced design in terms of sex, litter, and boar. Accordingly, out of the twenty-four sampled animals, twelve animals divergent in RFI values were selected for microarray analyses, producing six individual samples per RFI group (high RFI, low RFI). Total RNA was extracted from liver samples using TRI reagent (Sigma-Aldrich, Taufkirchen, Germany). Samples were treated with DNaseI (Roche, Mannheim, Germany) and purified with a column-based RNA extraction kit (NucleoSpin RNAII, Macherey-Nagel, Düren, Germany) according to manufacturer’s protocols. Quantity and purity were checked using a NanoDrop ND-1000 photospectrometer (NanoDrop, Peqlab, Erlangen, Germany). RNA integrity was assessed after separation of nucleic acids on agarose gels. No signs of a significant degradation of 28S and 18S bands were observed from agarose gels and all samples had 260/280 ratios above 2.1 indicating good RNA quality. Samples were checked for contamination by genomic DNA and showed no amplification of specific DNA targets.

### 4.5. Microarray Analysis

For the microarray experiment, biotin-labelled and fragmented single stranded cDNA was synthetized using an Affymetrix WT Plus Expression Kit (Affymetrix, Santa Clara, CA, USA). Pre-processed samples were subsequently hybridized to porcine *snowball* arrays (Affymetrix), which were designed to obtain individual genome-wide transcriptome profiles [47]. Processing of arrays followed manufacturer’s instructions using the GeneChip Hybridization, Wash, and Stain Kit (Affymetrix). After scanning, raw data were generated with Affymetrix GCOS 1.1.1 software (Affymetrix). Raw data have been deposited in a minimum information about a microarray experiment (MIAME)-compliant database, the National Center for Biotechnology Information Gene Expression Omnibus (Available online: www.ncbi.nlm.nih.gov/geo) (accession numbers: GSE95223).

### 4.6. Quantitative Real-Time PCR (RT-qPCR)

First-strand cDNA synthesis was carried out in a reaction with random primers, oligo d(T) 13VN, Superscript III reverse transcriptase (Invitrogen, Karlsruhe, Germany) and RNAsinPlus RNase Inhibitor (Promega, Heidelberg, Germany) using 1.5 µg of total RNA. The cDNA samples were diluted in 200 µL aqua dest. and stored at −20 °C until further analyses. Transcript levels of selected target (*ITGA5*, *NR1H4*, *SLC1A4*, *SLC7A9*, *SQLE*) and reference genes (*RPL10*, *RPL32*) were quantified by RT-qPCR. Individual mRNA samples (*n* = 12) were analyzed in duplicate on a LightCycler 480 system using the LightCycler 480 SYBR Green I Master (Roche, Mannheim, Germany) according to the manufacturer’s instructions. Reactions were carried out in a final volume of 12 µL and included 6.0 µL of LightCycler 480 SYBR Green I Master (Roche), 0.6 µL (10 µM) of each primer (Table S3), 2 µL cDNA, and 2.8 µL of Aqua dest. After an initial denaturation step at 95 °C for 10 min, 40 PCR cycles were performed including denaturation at 95 °C for 15 s, annealing for 10 s (temperatures as indicated in Table S3) and extension/fluorescence acquisition at 72 °C for 15 s. The absence of non-specific products was verified employing melting curve analyses and gel electrophoresis. Threshold cycles were converted to copy numbers using a standard curve made by amplifying serial dilutions of a corresponding reference amplificate (10^7^–10^2^ copies). *RPL10* and *RPL32* were used as housekeeping genes and correlations of normalized expression values were estimated using Spearman rank correlation (*n* = 12).

### 4.7. Phenotype Data Analyses

To compute the individual phenotypic observations (body weight, FE, physiological parameters, hormones, fatty acid profiles), a linear mixed-effect model was applied (R language, v3.2.3, package lme4). Data were weighted as a function of RFI values. Furthermore, random effects represented by dam and sire were included. The body weight related traits (liver weight, backfat) were corrected for live weight used as a covariate. Differences were considered significant at *p* < 0.05.

### 4.8. Transcript Data Analyses

The quality of microarray data was assessed using the R language for statistical computing v3.2.3 [48]. Raw intensity data were normalized using the Robust Multichip Average (RMA) approach. To improve statistical power [49], inappropriate probe sets were excluded from further analyses, i.e., probe sets with a low and high standard deviation (lower 10% quantile among all samples and upper 10% quantile within each experimental group). Differential expression was determined using the linear models for microarray analysis (limma) package within the R environment. To achieve the transcriptional contrast related to FE, the model included the RFI class as a fixed effect. Due to the balanced selection of RFI-divergent siblings of the same sex for microarray analysis, no further effects were considered. To account for multiple testing, *q*-values were calculated based on the *p*-value distribution using the FDR approach designed for large datasets [50]. The threshold level for q-values was set at *q* < 0.30, which corresponds to a *p*-value < 0.01. Fold changes displaying differences in mRNA abundances were calculated from least square means (positive FC: high-RFI < low-RFI, i.e., low-efficient animals < high-efficient animals; negative FC: high-RFI > low-RFI, i.e., low-efficient animals > high-efficient animals). The probe-set to gene symbol assignment was obtained from the developers of the *snowball* microarray [47]. For Ingenuity Pathway Analysis (IPA; Ingenuity Systems, Redwood City, CA, USA), orthologous human gene identifiers were retrieved using the BiomaRt R package. Pig and human genome assemblies were employed (*Sus Scrofa* 10.2 and GRCh38.p7). Lists of altered transcripts were evaluated with IPA in order to visualize canonical pathways and bio-functions. Data referring to RT-qPCR were factorial normalized and statistically analyzed as done for microarray data (RFI class as a fixed effect).

## 5. Conclusions

Taken together, the experimental data of the presumably-altered nutrient utilization in FE-divergent animals complements existing phenotypic findings on the molecular level. In particular, the hepatic profiles of fatty acids and expressed genes reveal that high-FE animals might favor metabolic shifts comprising lipid and carbohydrate utilization when compared to low-FE animals. The improvements in FE traits might be driven by intensified cellular infrastructure, as suggested by transcriptome data. Consequently, understanding physiologic requirements, such as processes mediating nutrient absorption, storage, and excretion will be essential to induce different and stable phenotypes towards an optimized nutrient supply and the highest standards of animal health and welfare in livestock production.

## Figures and Tables

**Table 1:** Phenotypic records obtained from animals divergent in feed efficiency (FE; measured by residual feed intake) at day 140 of life.

Item	Unit	High-FE (Mean ± SE)	Low-FE (Mean ± SE)	*p*-Value
Performance (*n* = 24)				
Body weight	kg	94.96 ± 2.14	91.13 ± 3.56	0.311
ADG (day 70–day 140)	kg/d	0.98 ± 0.02	0.94 ± 0.04	0.653
ADFI (day 70–day 140)	kg/d	1.90 ± 0.04	2.25 ± 0.06	<0.001
FCR (day 70–day 140)	kg/kg	1.94 ± 0.03	2.40 ± 0.06	<0.001
RFI	kg	−0.20 ± 0.02	0.21 ± 0.03	<0.001
Backfat	mm	4.78 ± 0.23	4.78 ± 0.31	0.650
Liver weight	kg	1.82 ± 0.08	1.65 ± 0.08	0.028
Blood parameters (*n* = 24)				
Albumin	g/dL	4.52 ± 0.11	4.24 ± 0.11	0.181
Glucose	mg/dL	145.67 ± 29.26	110.25 ± 12.05	0.183
GGT	U/L	49.83 ± 5.74	47.25 ± 3.99	0.600
GPT	U/L	41.50 ± 1.89	37.58 ± 2.07	0.175
GOT	U/L	49.08 ± 4.59	41.50 ± 3.39	0.175
Urea	mg/dL	10.89 ± 0.97	10.37 ± 0.44	0.155
LDH	U/L	179.42 ± 15.41	165.00 ± 15.19	0.408
Triglyceride	mg/dL	39.92 ± 5.58	24.75 ± 3.66	0.001
Total cholesterol	mg/dL	107.92 ± 5.45	107.83 ± 6.81	0.739
Total protein	g/dL	6.44 ± 0.11	6.56 ± 0.18	0.916
Amylase	U/L	828.17 ± 55.49	805.92 ± 55.36	0.553
Lipase	U/L	51.75 ± 7.05	50.08 ± 6.76	0.859
Hormones (*n* = 12)				
Triiodothyronine (T3)	ng/mL	0.59 ± 0.09	0.47 ± 0.06	0.269
Thyroxine (T4)	ng/mL	15.62 ± 1.60	15.67 ± 2.70	0.262

Associations exceeding significance threshold (*p* < 0.05) are highlighted in bold. ADG: average daily gain; ADFI: average daily feed intake; FCR: feed conversion ratio; RFI: residual feed intake; GGT: gamma-glutamyl transferase; GPT: glutamate pyruvate transaminase; GOT: glutamate oxaloacetate transaminase; LDH: lactate dehydrogenase.

**Table 2:** Fatty acid content in liver tissue obtained from animals divergent in feed efficiency (FE; measured by residual feed intake) at day 140 of life (*n* = 24). Values are displayed as mg/100 g liver.

Item	High-FE (Mean ± SE)	Low-FE (Mean ± SE)	*p*-Value
***Sum Fatty Acid Concentrations***			
Fat content (%)	2.36 ± 0.08	2.57 ± 0.09	<0.001
SFA ^1^	912.04 ± 31.63	994.21 ± 34.12	<0.001
MUFA ^2^	285.67 ± 10.68	315.63 ± 23.76	0.111
PUFA ^3^	1157.42 ± 49.23	1261.92 ± 44.00	<0.001
*n*-3 PUFA ^4^	151.04 ± 6.27	167.11 ± 6.77	0.007
*n*-6 PUFA ^5^	1006.38 ± 43.81	1094.81 ± 40.86	0.001
***Fatty Acid Concentrations***			
C10:0	2.05 ± 0.18	2.22 ± 0.23	0.375
C12:0	1.46 ± 0.08	1.58 ± 0.08	0.265
C13:0	0.40 ± 0.0	0.36 ± 0.02	0.028
***Fatty Acid Concentrations***			
C14:0	6.24 ± 0.29	7.08 ± 0.98	0.333
C15:0	4.26 ± 0.29	3.70 ± 0.26	0.029
C16:0	297.99 ± 10.10	328.30 ± 17.03	0.008
C17:0	30.49 ± 2.44	27.89 ± 2.57	0.627
C18:0	546.46 ± 27.46	597.87 ± 22.54	0.002
C16:1 *cis*-9	10.64 ± 0.82	12.32 ± 1.76	0.225
C18:1 *cis*-9	223.17 ± 8.75	247.07 ± 20.32	0.130
C18:1 *cis*-11	31.95 ± 1.60	33.16 ± 1.97	0.222
C18:1 *trans*-9	4.07 ± 0.22	4.20 ± 0.24	0.342
C18:1 *trans*-11	1.85 ± 0.05	1.79 ± 0.10	0.259
C20:1 *cis*-11	4.01 ± 0.18	4.22 ± 0.18	0.094
C18:2 *n*-6	397.90 ± 23.34	460.20 ± 26.03	<0.001
C18:3 *n*-3	10.25 ± 0.93	14.13 ± 2.29	0.059
C18:3 *n*-6	4.31 ± 0.19	5.24 ± 0.49	0.003
C20:2 *n*-6	12.55 ± 0.92	14.23 ± 0.78	0.011
C20:3 *n*-6	26.11 ± 2.19	29.58 ± 1.90	0.081
C20:4 *n*-6	535.06 ± 20.46	551.75 ± 32.88	0.264
C20:5 *n*-3	21.78 ± 1.64	26.20 ± 2.25	0.004
C22:4 *n*-6	29.56 ± 2.33	32.81 ± 1.64	0.144
C22:5 *n*-3	64.30 ± 3.61	67.75 ± 2.77	0.015
C22:6 *n*-3	50.03 ± 4.65	53.60 ± 4.67	0.324

^1^ Saturated fatty acid: sum of 10:0, 11:0, 12:0, 13:0, 14:0, 15:0, 16:0, 17:0, 18:0, 20:0, 21:0, 22:0, 23:0, 24:0; ^2^ Monounsaturated fatty acid: sum of 14:1, 15:1, 16:1, 17:1, 18:1*t*, 18:1*c*9, 18:1*c*11, 22:1, 24:1; ^3^ Polyunsaturated fatty acid: sum of 18:2*t*, 18:2*n*-6, 18:3*n*-3, 18:4*n*-3, 20:3*n*-6, 20:4*n*-6, 20:5*n*-3 22:4*n*-6, 22:5*n*-3, 22:6*n*-3, 18:2c9, *tr*11CLA, 18:3*n*-6, 20:2*n*-6, 20:3*n*-3, 22:2*n*-6; ^4^ *n*-3 PUFA: sum of 20:3*n*-3, 22:6*n*-3, 22:5*n*-3, 20:5*n*-3, 18:4*n*-3, 18:3*n*-3; ^5^ *n*-6 PUFA: sum of 22:2*n*-6, 20:2*n*-6, 18:3*n*-6, 22:4*n*-6, 20:3*n*-6, 18:2*n*-6, 20:4*n*-6; Associations exceeding significance threshold (*p* < 0.05) are highlighted in bold.

**Table 3:** Pathways altered between feed efficiency (FE; measured by residual feed intake)-divergent animals in liver tissue at a significance level of *p* < 0.01.

Regulated Pathway	Number of Genes	*p*-Value	Involved Genes (Fold Change) ^1^
Integrin Signaling	13	0.001	*ARF5* (+1.34), *ARHGAP5* (−1.34), *ARPC5L* (+1.36), *BRAF* (−1.37), *FYN* (−1.83), *ILK* (+1.41), *ITGA1* (−1.52), *ITGA5* (+1.61), *MYLK2* (+1.27), *PIK3C2A* (−1.37), *PIK3CB* (−1.49), *PPP1CB* (−1.36), *TSPAN6* (−1.48)
Ephrin A Signaling	6	0.002	*ADAM10* (−1.38), *EPHA5* (+1.3), *FYN* (−1.83), *PIK3C2A* (−1.37), *PIK3CB* (−1.49), *VAV3* (−1.55)
Adipogenesis pathway	9	0.002	*BMPR2* (−1.49), *CLOCK* (−1.65), *DDIT3* (+1.35), *FZD5* (−1.35), *GTF2H5* (−1.4), *HDAC2* (−1.31), *KLF3* (−1.34), *SIRT1* (−1.42), *TXNIP* (−1.75)
Insulin Receptor Signaling	8	0.010	*CBL* (−1.33), *FYN* (−1.83), *INSR* (−1.42), *PIK3C2A* (−1.37), *PIK3CB* (−1.49), *PPP1CB* (−1.36), *PRKAG2* (−1.33), *PTPRF* (+1.27)

^1^ Values in parentheses represent fold changes and indicate positive or negative transcript abundances (+: high-FE > low-FE; −: high-FE < low-FE).

**Table 4:** Significantly enriched bio-functions of carbohydrate, lipid, and amino acid metabolism.

Themes/Biofunctions	*p*-Value	Involved Genes (Fold Change) ^1^
***Carbohydrate Metabolism***		
Uptake of d-glucose	<0.001	*CBL* (−1.33), *CYLD* (−1.51), *DPP4* (−1.45), *EGLN3* (+1.32), *GNAS* (+1.65), *HGF* (−1.42), *IDH1* (−1.38), *INSR* (−1.42), *MYO1C* (+1.36), *PDK2* (+1.54), *PIK3C2A* (−1.37), *PIK3CB* (−1.49), *PPM1A* (−1.34), *PTPRF* (+1.27), *SIRT1* (−1.42), *TXNIP* (−1.75)
Quantity of glycogen	0.001	*GNAS* (+1.65), *IL6ST* (−1.38), *INSR* (−1.42), *LIFR* (−1.34), *NR1H4* (−1.34), *RPS6KA3* (−1.32), *SC5D* (−1.35), *XPA* (−1.39)
Uptake of carbohydrate	0.002	*CBL* (−1.33), *CYLD* (−1.51), *DPP4* (−1.45), *EGLN3* (+1.32), *GNAS* (+1.65), *HGF* (−1.42), *IDH1* (−1.38), *INSR* (−1.42), *MYO1C* (+1.36), *NR1H4* (−1.34), *PDK2* (+1.54), *PIK3C2A* (−1.37), *PIK3CB* (−1.49), *PPM1A* (−1.34), *PTPRF* (+1.27), *SIRT1* (−1.42), *TXNIP* (−1.75)
Oxidation of carbohydrate	0.003	*ESRRG* (+1.33), *INSR* (−1.42), *PDK2* (+1.54), *PNPLA8* (−1.34), *SIRT1* (−1.42)
Quantity of carbohydrate	0.010	*ESR1* (+1.36), *FOXA1* (+1.32), *GNAS* (+1.65), *GPR39* (+1.39), *HGF* (−1.42), *IL6ST* (−1.38), *INSR* (−1.42), *ITPR2* (−1.32), *KDM3A* (−1.46), *LIFR* (−1.34), *NR1H4* (−1.34), *PNPLA8* (−1.34), *PSEN2* (−1.39), *RPS6KA3* (−1.32), *SC5D* (−1.35), *SGMS2* (−1.50), *SIRT1* (−1.42), *SLC25A13* (+1.30), *SLC3A2* (+1.39), *STEAP3* (+1.37), *TXNIP* (−1.75), *VPS13C* (−1.33), *XPA* (−1.39)
Disposal of d-glucose	0.010	*INSR* (−1.42), *NR1H4* (−1.34), *PTPRF* (+1.27)
Oxidation of d-glucose	0.012	*ESRRG* (+1.33), *INSR* (−1.42), *PDK2* (+1.54), *PNPLA8* (−1.34)
Phosphorylation of phosphatidylinositol	0.015	*FAM126A* (−1.37), *PIK3C2A* (−1.37), *PIK3CB* (−1.49)
Import of carbohydrate	0.019	*B4GALT1* (+1.35), *ESR1* (+1.36), *INSR* (−1.42), *PRKAG2* (−1.33), *TXNIP* (−1.75)
***Lipid Metabolism***		
Synthesis of steroid	0.004	*ACAT1* (−1.31), *BMPR2* (−1.49), *ESR1* (+1.36), *FOXA1* (+1.32), *HGF* (−1.42), *NR1H4* (−1.34), *PDE8A* (−1.31), *PRKAG2* (−1.33), *SIRT1* (−1.42), *SLC9A3R2* (+1.33), *TLR3* (−1.34), *TLR4* (−1.40), *TRERF1* (−1.37)
Steroidogenesis of cells	0.004	*SLC9A3R2* (+1.33), *TLR3* (−1.34), *TLR4* (−1.40)
Synthesis of thromboxane	0.005	*NTN1* (+1.32), *PIK3CB* (−1.49), *PNPLA8* (−1.34)
Concentration of fatty acid	0.006	*CBL* (−1.33), *GNAS* (+1.65), *IDH1* (−1.38), *INSR* (−1.42), *ITGA1* (−1.43), *KDM3A* (−1.46), *NR1H4* (−1.34), *NTN1* (+1.32), *PNPLA8* (−1.34), *SIRT1* (−1.42), *SLC25A13* (+1.30), *SNRK* (−1.35), *TXNIP* (−1.75), *XPA* (−1.39)
Concentration of acylglycerol	0.016	*CBL* (−1.33), *FOXA1* (+1.32), *GNAS* (+1.65), *HGF* (−1.42), *IDH1* (−1.38), *INSR* (−1.42), *ITGA1* (−1.43), *KDM3A* (−1.46), *NR1H4* (−1.34), *PDK2* (+1.54), *PNPLA8* (−1.34), *SGMS2* (−1.5), *SIRT1* (−1.42), *SLC25A13* (+1.30), *SNRK* (−1.35), *TXNIP* (−1.75)
Concentration of triacylglycerol	0.017	*CBL* (−1.33), *FOXA1* (+1.32), *GNAS* (+1.65), *HGF* (−1.42), *IDH1* (−1.38), *INSR* (−1.42), *ITGA1* (−1.43), *KDM3A* (−1.46), *NR1H4* (−1.34), *PDK2* (+1.54), *PNPLA8* (−1.34), *SIRT1* (−1.42), *SLC25A13* (+1.30), *SNRK* (−1.35), *TXNIP* (−1.75)
***Amino Acid Metabolism***		
Transport of neutral amino acid	0.013	*SLC1A4* (−1.66), *SLC3A2* (+1.39), *SLC7A9* (+1.50)

^1^ Values in parentheses represent fold changes and indicate positive or negative transcript abundances (+: high-FE > low-FE; −: high-FE < low-FE). Bio-functions represented by less than three molecules were excluded.

**Table 5:** Comparison of microarray and quantitative real-time PCR (RT-qPCR) results for selected transcripts to verify microarray data; Fold change (FC).

Transcript	Microarray			RT-qPCR			Correlation	
Gene Symbol	Probe-Set ID	FC ^1^	*p*-Value	*q*-Value	FC ^1^	*p*-Value	Coefficient	*p*-Value
*ITGA5*	SNOWBALL_006991	+1.61	<0.001	0.028	+1.82	0.011	0.94	<0.001
*NR1H4*	SNOWBALL_007505	−1.34	0.002	0.181	−1.29	0.048	0.69	0.013
*SLC1A4*	SNOWBALL_005484	−1.66	<0.001	0.022	−1.69	0.003	0.91	<0.001
*SLC7A9*	SNOWBALL_026778	+1.87	<0.001	0.010	+1.87	0.017	0.85	0.001
*SQLE*	SNOWBALL_000764	−1.82	<0.001	0.004	−1.89	0.005	0.91	<0.001

^1^ Fold change (+: high-FE > low-FE; −: high-FE < low-FE).

## Supplementary Materials

Supplementary materials can be found at www.mdpi.com/1422-0067/18/8/1674/s1.

## Abbreviations

FE	Feed efficiency
RFI	Residual feed intake
FCR	Feed conversion ratio
ADG	Average daily weight gain
ADFI	Average daily feed intake
SFA	Saturated fatty acids
MUFA	Monounsaturated fatty acids
PUFA	Polyunsaturated fatty acids
