# Supplementary material for: Strategies towards Improved Feed Efficiency in Pigs Comprise Molecular Shifts in Hepatic Lipid and Carbohydrate Metabolism
Source: Int J Mol Sci. 2017 Aug 1;18(8):1674. doi: 10.3390/ijms18081674 (PMC5578064; doi:10.3390/ijms18081674)
Supplement: Supplementary file 1 [file ijms-18-01674-s001.pdf]

Supplementary Table S1. Probe-set IDs, fold changes, average expression values, and statistics of significantly altered transcripts (see Materials and Methods section for details)

Probeset	Fold change (FC)	AveExpr	p -value	q-value	SSC10 gene annotation
SNOWBALL_000763	-2.13	4.788	2.56E-09	8.06E-05	SQLE
bta-mir-2378	-1.99	4.183	7.27E-07	0.008	
SNOWBALL_000034	-1.83	4.472	1.09E-06	0.009	FYN
SNOWBALL_000764	-1.82	9.267	2.71E-07	0.004	SQLE
SNOWBALL_029065	-1.81	4.275	3.68E-06	0.014	SLC30A10
SNOWBALL_006620	-1.75	9.505	6.71E-06	0.014	TXNIP
SNOWBALL_030137	-1.74	3.768	2.75E-05	0.030	CREBRF
SNOWBALL_025999	-1.70	3.742	3.61E-06	0.014	ARHGAP35
SNOWBALL_034367	-1.69	4.131	1.46E-05	0.022	BCLAF1
SNOWBALL_023165	-1.68	9.074	1.03E-05	0.020	SQLE
SNOWBALL_007178	-1.67	4.925	4.26E-06	0.014	TBC1D15
SNOWBALL_042886	-1.66	4.124	5.41E-05	0.039	PEX1
SNOWBALL_005484	-1.66	5.673	1.52E-05	0.022	SLC1A4
SNOWBALL_011532	-1.65	6.481	4.45E-06	0.014	CLOCK
SNOWBALL_007751	-1.65	5.907	3.61E-05	0.032	EEA1
SNOWBALL_018580	-1.65	5.780	1.96E-06	0.010	LMBRD2
SNOWBALL_031722	-1.64	5.706	2.35E-05	0.027	LRRC8B
SNOWBALL_005073	-1.63	6.240	2.83E-05	0.030	PPP4R3B
SNOWBALL_006396	-1.61	6.054	5.31E-05	0.039	STXBP3
SNOWBALL_017161	-1.60	6.338	5.55E-05	0.039	JMJD1C
SNOWBALL_000426	-1.60	4.515	1.31E-05	0.022	NNAT
SNOWBALL_002802	-1.60	5.171	2.93E-05	0.030	PRPF39
SNOWBALL_028963	-1.59	4.437	6.70E-05	0.042	
SNOWBALL_023524	-1.59	4.684	1.55E-05	0.022	STAG2
SNOWBALL_004810	-1.59	6.081	6.41E-06	0.014	ROGDI
SNOWBALL_017906	-1.59	7.135	8.51E-05	0.044	RBMS1
SNOWBALL_034131	-1.59	6.500	6.56E-05	0.042	ENSSSCG00000025315
SNOWBALL_018629	-1.58	4.999	6.74E-05	0.042	RICTOR
SNOWBALL_009254	-1.57	5.793	7.41E-05	0.044	ENSSSCG00000003727
SNOWBALL_003094	-1.57	7.141	0.0002	0.066	PHLPP1
SNOWBALL_009377	-1.57	6.405	1.12E-05	0.020	ENSSSCG00000003728
SNOWBALL_007306	-1.57	6.431	0.0002	0.069	LOC102158163
SNOWBALL_020919	-1.56	5.666	3.11E-05	0.030	BIRC3
SNOWBALL_017205	-1.56	2.161	0.0001	0.053	TBC1D12
SNOWBALL_011147	-1.56	1.711	0.0003	0.083	ENSSSCG00000019731
SNOWBALL_000070	-1.56	3.492	7.11E-05	0.044	
SNOWBALL_005267	-1.56	4.290	3.52E-05	0.032	ENSSSCG00000008591
SNOWBALL_010165	-1.56	6.159	9.89E-05	0.046	ATG2B
SNOWBALL_000694	-1.56	4.240	4.61E-05	0.038	REL
SNOWBALL_025782	-1.55	2.182	0.0002	0.064	
mmu-mir-142	-1.55	1.755	8.50E-05	0.044	MIR142
SNOWBALL_006244	-1.55	4.540	0.0001	0.057	ENSSSCG00000006851
SNOWBALL_027882	-1.55	4.963	0.0001	0.057	ENSSSCG00000023253
SNOWBALL_034189	-1.55	2.371	7.99E-05	0.044	
SNOWBALL_029772	-1.54	3.960	6.42E-05	0.042	MYNN
SNOWBALL_009068	-1.54	3.953	3.32E-05	0.031	FPGT

SNOWBALL_001800	-1.54	2.879	0.0005	0.102	TBC1D8B
mmu-mir-181b-2	-1.54	1.846	0.0001	0.054	
SNOWBALL_033185	-1.54	3.702	0.0003	0.081	TNPO1
hsa-mir-3135	-1.53	5.081	0.0002	0.069	
SNOWBALL_022869	-1.53	4.215	5.54E-05	0.039	CYP2C91
SNOWBALL_011377	-1.53	5.371	7.90E-05	0.044	TLR1
SNOWBALL_040179	-1.53	2.811	8.42E-05	0.044	MAP3K2
SNOWBALL_045390	-1.52	2.911	0.0001	0.056	N4BP2
SNOWBALL_026406	-1.52	7.275	0.0003	0.083	TMEM59
SNOWBALL_045434	-1.52	2.202	0.0002	0.069	
SNOWBALL_003169	-1.52	7.456	1.83E-05	0.024	ZFAND5
SNOWBALL_004607	-1.52	7.064	0.0002	0.069	RMND5A
SNOWBALL_028279	-1.52	4.639	8.98E-05	0.045	ITGA1
SNOWBALL_041464	-1.52	5.641	0.0004	0.099	PEX1
SNOWBALL_019112	-1.52	5.429	0.0001	0.054	GPCPD1
SNOWBALL_044159	-1.51	2.287	0.0004	0.089	
SNOWBALL_003260	-1.51	5.975	0.0007	0.117	COPS2
SNOWBALL_046470	-1.51	3.290	0.0005	0.101	
SNOWBALL_000598	-1.51	5.756	0.0002	0.069	TAX1BP1
SNOWBALL_008133	-1.51	4.486	0.0003	0.081	CYLD
SNOWBALL_019513	-1.51	2.922	0.0003	0.086	ENSSSCG00000019914
SNOWBALL_002507	-1.51	6.692	7.78E-05	0.044	CDK19
SNOWBALL_020553	-1.51	5.269	0.0003	0.087	STAG2
SNOWBALL_017840	-1.51	8.440	0.0005	0.104	UPP2
SNOWBALL_028176	-1.50	5.211	0.0010	0.138	TM9SF3
SNOWBALL_015487	-1.50	4.039	0.0003	0.082	TTC14
SNOWBALL_031997	-1.50	2.677	0.0001	0.053	
SNOWBALL_018690	-1.50	6.948	0.0003	0.086	CPEB4
SNOWBALL_011947	-1.50	4.439	0.0006	0.109	SGMS2
SNOWBALL_026583	-1.50	3.892	7.54E-05	0.044	NPL
SNOWBALL_009148	-1.50	4.441	0.0006	0.113	DNAJB4
SNOWBALL_033090	-1.50	5.816	0.0004	0.092	MTFR1
SNOWBALL_034677	-1.50	4.345	0.0003	0.083	
SNOWBALL_034970	-1.50	4.059	0.0008	0.120	ZNF638
SNOWBALL_023332	-1.49	6.271	0.0005	0.101	ATXN3
SNOWBALL_015991	-1.49	8.885	0.0002	0.069	CLDND1
SNOWBALL_031440	-1.49	5.933	3.89E-05	0.033	ENSSSCG00000021203
SNOWBALL_032740	-1.49	4.054	0.0003	0.087	
SNOWBALL_029908	-1.49	5.473	0.0005	0.101	BMPR2
SNOWBALL_012978	-1.49	5.016	0.0002	0.068	C9H1orf27
SNOWBALL_008731	-1.49	5.875	0.0002	0.072	AGO4
SNOWBALL_020406	-1.48	5.535	0.0011	0.145	TSPAN6
SNOWBALL_026989	-1.48	2.721	0.0002	0.065	RSRC1
SNOWBALL_026521	-1.48	5.444	0.0005	0.101	DPY19L1
SNOWBALL_017354	-1.48	6.063	0.0007	0.115	CCSER2
SNOWBALL_018101	-1.48	8.470	8.53E-05	0.044	MYO1B
bta-mir-2308	-1.48	4.404	0.0018	0.175	
SNOWBALL_000369	-1.48	8.228	0.0010	0.138	DPP4
SNOWBALL_009212	-1.48	5.556	0.0001	0.059	RNF138
SNOWBALL_035136	-1.48	5.714	0.0003	0.081	

SNOWBALL_013031	-1.48	2.356	0.0007	0.115	ENSSSCG00000020013
SNOWBALL_020932	-1.48	7.435	0.0002	0.069	
SNOWBALL_013821	-1.48	5.398	0.0003	0.081	INTS6
SNOWBALL_010455	-1.48	4.510	0.0007	0.117	ZBTB1
SNOWBALL_031764	-1.47	2.621	4.91E-05	0.039	
SNOWBALL_014731	-1.47	5.666	0.0006	0.111	
SNOWBALL_011711	-1.47	3.325	0.0002	0.069	C8H4orf32
SNOWBALL_021578	-1.47	3.869	5.27E-05	0.039	
SNOWBALL_009684	-1.47	4.421	0.0007	0.116	GMNN
SNOWBALL_000374	-1.46	7.018	0.0007	0.117	
SNOWBALL_004956	-1.46	7.055	0.0002	0.065	PUM2
SNOWBALL_016912	-1.46	5.251	0.0004	0.092	ENSSSCG00000010491
SNOWBALL_004901	-1.46	3.515	0.0002	0.072	KDM3A
SNOWBALL_028230	-1.46	2.677	0.0018	0.175	SNRK
SNOWBALL_013457	-1.46	8.599	0.0008	0.120	MRC1
SNOWBALL_032188	-1.46	3.445	0.0005	0.101	
SNOWBALL_011379	-1.46	5.332	0.0010	0.137	FRYL
SNOWBALL_035411	-1.46	5.242	0.0008	0.120	
SNOWBALL_047469	-1.46	3.962	0.0003	0.083	
SNOWBALL_006364	-1.46	6.834	0.0005	0.101	ARHGAP29
SNOWBALL_026437	-1.46	2.580	0.0014	0.160	
SNOWBALL_021612	-1.46	5.262	0.0008	0.119	CWF19L2
SNOWBALL_044937	-1.45	5.475	0.0008	0.121	HIPK3
SNOWBALL_022021	-1.45	6.103	0.0023	0.194	RPF1
SNOWBALL_013977	-1.45	4.210	0.0010	0.138	GPR183
SNOWBALL_000370	-1.45	8.784	0.0017	0.175	DPP4
mmu-mir-1969	-1.45	1.537	0.0018	0.175	
SNOWBALL_003666	-1.45	5.066	0.0009	0.125	GNA14
SNOWBALL_028568	-1.45	3.417	0.0015	0.163	GPATCH2L
SNOWBALL_018686	-1.45	6.172	0.0012	0.149	ERBB2IP
SNOWBALL_027704	-1.45	5.035	0.0031	0.217	
SNOWBALL_046629	-1.44	2.672	0.0009	0.131	
SNOWBALL_017226	-1.44	4.289	0.0005	0.101	CCDC186
SNOWBALL_025225	-1.44	3.788	0.0012	0.152	USP15
SNOWBALL_038001	-1.44	3.936	0.0022	0.189	FAM126A
SNOWBALL_036094	-1.44	5.847	0.0018	0.175	MED13L
SNOWBALL_003664	-1.44	5.785	0.0004	0.096	
SNOWBALL_010349	-1.44	5.150	0.0002	0.069	ENSSSCG00000002499
SNOWBALL_002314	-1.44	6.437	0.0005	0.104	
SNOWBALL_009235	-1.44	4.940	0.0003	0.087	ANKRD12
SNOWBALL_024040	-1.44	3.987	0.0011	0.144	ENSSSCG00000016291
SNOWBALL_013012	-1.44	7.339	0.0008	0.118	RASSF5
bta-mir-10b	-1.44	1.530	0.0007	0.117	MIR10B
SNOWBALL_030638	-1.44	5.833	0.0017	0.175	LOC106504717
SNOWBALL_028676	-1.44	7.245	0.0007	0.117	LMAN1
SNOWBALL_011336	-1.44	5.563	0.0008	0.121	N4BP2
SNOWBALL_017680	-1.43	5.036	0.0010	0.138	CCNT2
SNOWBALL_039501	-1.43	2.738	0.0005	0.101	LOC106509177
SNOWBALL_029661	-1.43	2.710	0.0012	0.147	
SNOWBALL_034579	-1.43	5.449	0.0006	0.115	ITGA1

SNOWBALL_047632	-1.43	5.723	0.0004	0.096	
SNOWBALL_004610	-1.43	5.560	0.0003	0.081	ANAPC1
SNOWBALL_032571	-1.43	4.049	0.0024	0.199	
SNOWBALL_026469	-1.43	6.513	0.0007	0.116	ZNF638
SNOWBALL_002495	-1.43	4.936	0.0003	0.086	ICE2
SNOWBALL_024675	-1.43	7.416	0.0022	0.192	VPS26A
SNOWBALL_035308	-1.43	3.160	0.0007	0.116	CNOT8
SNOWBALL_003925	-1.43	1.834	0.0036	0.227	
SNOWBALL_012868	-1.42	6.104	0.0019	0.177	HGF
SNOWBALL_002285	-1.42	4.246	0.0006	0.114	ZNF292
SNOWBALL_018543	-1.42	7.360	0.0007	0.115	
SNOWBALL_019754	-1.42	6.045	0.0007	0.115	ENSSSCG00000016648
SNOWBALL_039461	-1.42	7.015	0.0013	0.158	PEX1
SNOWBALL_028050	-1.42	6.712	0.0030	0.215	DPY19L3
SNOWBALL_042898	-1.42	4.688	0.0003	0.078	
SNOWBALL_019906	-1.42	7.069	0.0009	0.125	
SNOWBALL_011738	-1.42	4.049	0.0011	0.145	DNAJB14
SNOWBALL_024488	-1.42	5.593	0.0012	0.149	REEP3
SNOWBALL_017486	-1.42	5.499	0.0017	0.173	SIRT1
SNOWBALL_000197	-1.42	5.528	0.0010	0.138	DHX40
SNOWBALL_025746	-1.42	6.851	0.0008	0.121	RASSF5
SNOWBALL_012961	-1.42	5.844	0.0003	0.087	ENSSSCG00000023253
SNOWBALL_027575	-1.42	3.979	0.0007	0.115	
SNOWBALL_043148	-1.42	2.264	0.0023	0.196	
SNOWBALL_031964	-1.42	7.548	0.0007	0.116	NEBL
SNOWBALL_028242	-1.42	7.353	0.0020	0.181	INSR
SNOWBALL_027652	-1.41	3.576	0.0018	0.175	TMEM199
SNOWBALL_024816	-1.41	4.814	0.0026	0.205	SMC3
hsa-mir-614	-1.41	5.484	0.0014	0.162	
SNOWBALL_035490	-1.41	3.384	0.0005	0.102	
SNOWBALL_028248	-1.41	1.867	0.0004	0.099	
SNOWBALL_025858	-1.41	6.568	0.0037	0.234	
SNOWBALL_002586	-1.41	4.580	0.0014	0.160	ENSSSCG00000004358
SNOWBALL_045321	-1.41	6.504	0.0008	0.121	IL6ST
SNOWBALL_000093	-1.41	5.438	0.0004	0.099	CD72
SNOWBALL_040550	-1.41	2.068	0.0008	0.121	
SNOWBALL_013354	-1.41	4.850	0.0049	0.257	ENSSSCG00000010907
SNOWBALL_015023	-1.41	6.183	0.0007	0.117	ZFP3
SNOWBALL_033393	-1.41	2.934	0.0017	0.175	
SNOWBALL_002095	-1.41	6.669	0.0005	0.100	BCLAF1
SNOWBALL_001672	-1.41	5.204	0.0007	0.116	CXHXorf21
SNOWBALL_004202	-1.41	4.224	0.0020	0.182	TRPM6
SNOWBALL_017661	-1.41	4.442	0.0016	0.167	EPC2
SNOWBALL_046734	-1.41	5.645	0.0007	0.115	
SNOWBALL_046577	-1.40	2.971	0.0012	0.147	
SNOWBALL_005309	-1.40	1.451	0.0005	0.102	ENSSSCG00000018775
SNOWBALL_004987	-1.40	5.799	0.0019	0.179	PPM1B
SNOWBALL_013824	-1.40	4.278	0.0003	0.087	LRCH1
SNOWBALL_018997	-1.40	5.333	0.0007	0.115	FRG1
SNOWBALL_034752	-1.40	5.011	0.0024	0.199	LOC100626645

SNOWBALL_017930	-1.40	6.288	0.0009	0.124	
SNOWBALL_012682	-1.40	4.957	0.0015	0.162	SOX13
SNOWBALL_020228	-1.40	4.806	0.0038	0.234	GTF2H5
SNOWBALL_018187	-1.40	5.597	0.0014	0.160	BZW1
SNOWBALL_017025	-1.40	5.280	0.0005	0.102	TSNAX
SNOWBALL_046402	-1.40	3.854	0.0014	0.162	UBXN7
SNOWBALL_027356	-1.40	4.506	0.0009	0.123	
SNOWBALL_013359	-1.40	6.058	0.0010	0.138	LOC100520324
SNOWBALL_024499	-1.40	5.984	0.0005	0.101	ARID5B
SNOWBALL_024813	-1.40	5.054	0.0009	0.123	SHOC2
SNOWBALL_012905	-1.40	1.819	0.0033	0.222	ENSSSCG00000018984
SNOWBALL_009919	-1.40	8.736	0.0013	0.155	MUT
SNOWBALL_003302	-1.40	6.252	0.0014	0.160	TMEM87A
SNOWBALL_027878	-1.40	4.468	0.0048	0.255	ENSSSCG00000031028
SNOWBALL_000107	-1.40	5.559	0.0020	0.181	TLR4
SNOWBALL_038086	-1.40	3.700	0.0007	0.116	
SNOWBALL_027864	-1.39	4.894	0.0030	0.215	
SNOWBALL_021036	-1.39	6.943	0.0041	0.241	SLC30A1
SNOWBALL_013444	-1.39	5.912	0.0007	0.117	TOPORS
SNOWBALL_036966	-1.39	3.705	0.0026	0.203	PSMA2
SNOWBALL_009066	-1.39	4.675	0.0027	0.205	SSX2IP
SNOWBALL_013277	-1.39	9.285	0.0057	0.268	PSEN2
SNOWBALL_026358	-1.39	3.934	0.0025	0.200	
SNOWBALL_007651	-1.39	4.108	0.0021	0.182	NTN4
SNOWBALL_042373	-1.39	2.671	0.0039	0.236	FOXP1
SNOWBALL_019695	-1.39	6.532	0.0055	0.266	
SNOWBALL_020073	-1.39	4.418	0.0011	0.144	SCML1
SNOWBALL_019143	-1.39	6.365	0.0039	0.236	EIF2S2
SNOWBALL_035593	-1.39	4.143	0.0042	0.242	RYBP
SNOWBALL_010731	-1.39	7.926	0.0025	0.200	RDH11
SNOWBALL_027951	-1.39	2.339	0.0018	0.176	CNTN4
SNOWBALL_047556	-1.39	4.378	0.0006	0.112	C9H1orf27
SNOWBALL_003595	-1.39	4.455	0.0030	0.215	XPA
SNOWBALL_040006	-1.39	4.106	0.0015	0.163	RALGPS2
SNOWBALL_033713	-1.39	2.409	0.0016	0.167	
SNOWBALL_024489	-1.39	4.291	0.0026	0.203	JMJD1C
SNOWBALL_015477	-1.39	5.252	0.0042	0.242	LOC106505651
SNOWBALL_006291	-1.39	4.521	0.0034	0.225	LOC100157359
SNOWBALL_046733	-1.39	2.235	0.0017	0.175	
SNOWBALL_035345	-1.38	2.543	0.0032	0.220	ZFHX4
mmu-mir-466f-4	-1.38	2.324	0.0025	0.200	
SNOWBALL_033879	-1.38	5.459	0.0017	0.172	MYO1B
SNOWBALL_044870	-1.38	2.779	0.0007	0.117	
SNOWBALL_022761	-1.38	10.184	0.0050	0.257	LOC100523371
SNOWBALL_007539	-1.38	6.701	0.0018	0.175	KIAA1033
SNOWBALL_002897	-1.38	6.826	0.0030	0.215	TRPM7
SNOWBALL_003242	-1.38	2.982	0.0030	0.215	CDKL1
SNOWBALL_006062	-1.38	8.517	0.0040	0.240	TTPA
SNOWBALL_031582	-1.38	5.316	0.0050	0.257	
SNOWBALL_034120	-1.38	4.026	0.0021	0.188	LOC106510465

SNOWBALL_013302	-1.38	6.661	0.0008	0.122	LOC106504096
SNOWBALL_035110	-1.38	8.136	0.0076	0.300	CALCOCO1
SNOWBALL_000388	-1.38	8.086	0.0016	0.171	IL6ST
SNOWBALL_024676	-1.38	5.309	0.0022	0.189	SRGN
SNOWBALL_002391	-1.38	4.339	0.0015	0.164	ADAM10
SNOWBALL_040572	-1.38	2.356	0.0017	0.173	
SNOWBALL_031058	-1.38	3.367	0.0027	0.205	MPP5
SNOWBALL_021805	-1.38	4.805	0.0052	0.261	SENP6
SNOWBALL_005147	-1.38	5.708	0.0017	0.175	ENSSSCG00000008382
SNOWBALL_027843	-1.38	3.289	0.0018	0.175	LOC106507988
SNOWBALL_023996	-1.38	9.146	0.0025	0.200	IDH1
SNOWBALL_031750	-1.38	4.079	0.0025	0.200	LOC100738764
SNOWBALL_021653	-1.37	4.495	0.0028	0.207	
hsa-mir-3156-1	-1.37	6.892	0.0028	0.210	
hsa-mir-1248	-1.37	1.261	0.0020	0.182	
SNOWBALL_031583	-1.37	3.035	0.0039	0.236	BRAF
SNOWBALL_009207	-1.37	2.391	0.0029	0.212	STIL
SNOWBALL_008800	-1.37	6.739	0.0014	0.161	ADGRL4
SNOWBALL_013282	-1.37	7.631	0.0047	0.249	PSEN2
SNOWBALL_018081	-1.37	10.737	0.0020	0.182	
SNOWBALL_012554	-1.37	6.362	0.0030	0.215	FAM126A
SNOWBALL_034980	-1.37	4.218	0.0041	0.241	
SNOWBALL_002207	-1.37	6.872	0.0046	0.249	RNF217
hsa-mir-1283-1	-1.37	1.405	0.0019	0.177	
SNOWBALL_003318	-1.37	5.806	0.0011	0.145	TMX3
SNOWBALL_021020	-1.37	6.815	0.0056	0.266	SEC62
SNOWBALL_047030	-1.37	7.391	0.0042	0.241	LOC106504717
SNOWBALL_032076	-1.37	5.171	0.0022	0.192	ELL2
SNOWBALL_030103	-1.37	2.997	0.0038	0.234	
SNOWBALL_002380	-1.37	6.292	0.0019	0.177	CCPG1
SNOWBALL_038040	-1.37	4.489	0.0018	0.175	FCHO2
SNOWBALL_034041	-1.37	4.884	0.0006	0.115	
SNOWBALL_010533	-1.37	7.234	0.0064	0.279	PTGR2
SNOWBALL_027450	-1.37	3.627	0.0052	0.261	MBTD1
SNOWBALL_008956	-1.37	5.992	0.0016	0.171	DOCK7
SNOWBALL_010078	-1.37	4.669	0.0036	0.228	TRERF1
SNOWBALL_002374	-1.37	4.840	0.0025	0.200	LOC100517018
SNOWBALL_005046	-1.37	7.052	0.0029	0.213	EML4
SNOWBALL_001412	-1.37	3.525	0.0037	0.230	HLA-DMA
SNOWBALL_007331	-1.37	5.144	0.0018	0.175	PARP11
SNOWBALL_025736	-1.37	5.835	0.0024	0.197	ITSN2
SNOWBALL_025472	-1.37	6.272	0.0052	0.261	INTS12
SNOWBALL_003473	-1.37	2.927	0.0042	0.241	
SNOWBALL_016723	-1.37	2.556	0.0028	0.207	SRRD
SNOWBALL_009264	-1.37	4.921	0.0056	0.268	HOOK1
SNOWBALL_012620	-1.37	3.230	0.0069	0.283	CRTAM
SNOWBALL_005104	-1.37	1.536	0.0051	0.259	ENSSSCG00000019079
SNOWBALL_006849	-1.37	3.241	0.0011	0.145	MTERF2
SNOWBALL_047051	-1.37	5.340	0.0033	0.222	PIK3C2A
SNOWBALL_026365	-1.37	8.121	0.0037	0.230	LOC100522498

SNOWBALL_021635	-1.37	5.766	0.0055	0.266	
SNOWBALL_034775	-1.36	3.115	0.0020	0.182	
mmu-mir-297c	-1.36	1.567	0.0028	0.210	
SNOWBALL_013813	-1.36	3.951	0.0027	0.205	LPAR6
SNOWBALL_039564	-1.36	2.915	0.0027	0.205	
SNOWBALL_027872	-1.36	2.916	0.0057	0.268	
SNOWBALL_043342	-1.36	4.666	0.0011	0.144	PRELID2
SNOWBALL_009561	-1.36	6.945	0.0057	0.268	DEK
SNOWBALL_031712	-1.36	6.477	0.0049	0.257	
bta-mir-2370	-1.36	4.044	0.0017	0.173	
SNOWBALL_028427	-1.36	3.106	0.0023	0.196	
SNOWBALL_024837	-1.36	2.970	0.0049	0.257	OBFC1
SNOWBALL_005779	-1.36	6.722	0.0022	0.188	RNF19A
SNOWBALL_038200	-1.36	3.970	0.0007	0.116	
SNOWBALL_018976	-1.36	6.726	0.0012	0.149	CNOT7
SNOWBALL_018825	-1.36	5.196	0.0031	0.217	SLU7
SNOWBALL_004964	-1.36	6.367	0.0025	0.201	PPP1CB
SNOWBALL_013274	-1.36	4.327	0.0050	0.257	ENSSSCG00000010871
SNOWBALL_037501	-1.36	3.157	0.0073	0.293	ENSSSCG00000021742
SNOWBALL_018183	-1.36	4.391	0.0019	0.179	KIAA1715
SNOWBALL_039264	-1.36	1.912	0.0021	0.182	
SNOWBALL_015636	-1.36	3.383	0.0009	0.131	TMF1
SNOWBALL_000828	-1.36	5.593	0.0014	0.162	ARFGEF1
SNOWBALL_018823	-1.36	3.298	0.0041	0.241	ADAMTS6
SNOWBALL_015754	-1.36	6.200	0.0021	0.186	NAALADL2
bta-mir-2397	-1.36	1.720	0.0067	0.281	
SNOWBALL_013602	-1.36	2.043	0.0054	0.264	MIR27B
SNOWBALL_011837	-1.36	4.238	0.0008	0.118	FAM175A
SNOWBALL_037371	-1.36	4.581	0.0033	0.222	
SNOWBALL_025669	-1.36	6.434	0.0016	0.167	RB1CC1
SNOWBALL_033269	-1.36	1.570	0.0024	0.198	
SNOWBALL_037612	-1.36	3.884	0.0058	0.269	RC3H2
SNOWBALL_013308	-1.36	3.229	0.0019	0.177	ERCC6L2
SNOWBALL_033563	-1.36	7.227	0.0050	0.257	LOC106504520
SNOWBALL_020454	-1.36	6.900	0.0041	0.241	UTP14A
SNOWBALL_037010	-1.36	3.699	0.0021	0.182	LACE1
SNOWBALL_032675	-1.36	2.539	0.0030	0.215	
SNOWBALL_012813	-1.35	3.922	0.0054	0.264	KRIT1
SNOWBALL_015261	-1.35	5.897	0.0018	0.175	SNRK
SNOWBALL_035298	-1.35	2.570	0.0017	0.173	
SNOWBALL_024683	-1.35	4.895	0.0022	0.192	
SNOWBALL_011394	-1.35	4.929	0.0043	0.243	WDR19
SNOWBALL_028942	-1.35	4.498	0.0021	0.182	LRP11
SNOWBALL_029873	-1.35	2.606	0.0059	0.270	LOC100156637
SNOWBALL_020820	-1.35	2.946	0.0058	0.269	MIR105-2
SNOWBALL_018032	-1.35	7.792	0.0042	0.241	FZD5
SNOWBALL_019000	-1.35	5.752	0.0050	0.257	ENSSSCG00000006993
SNOWBALL_035373	-1.35	6.109	0.0015	0.163	
SNOWBALL_019682	-1.35	5.320	0.0036	0.229	KDM7A
SNOWBALL_026489	-1.35	3.540	0.0038	0.235	RNMT

SNOWBALL_046363	-1.35	1.887	0.0026	0.202	
SNOWBALL_006513	-1.35	5.497	0.0024	0.199	ARHGAP29
SNOWBALL_012463	-1.35	6.517	0.0052	0.260	ARHGAP42
SNOWBALL_024549	-1.35	3.836	0.0044	0.244	TSNAX
SNOWBALL_013076	-1.35	4.827	0.0039	0.236	SRGAP2
SNOWBALL_004642	-1.35	4.140	0.0033	0.222	GCC2
SNOWBALL_025848	-1.35	5.103	0.0028	0.207	CNIH
SNOWBALL_016431	-1.35	4.399	0.0014	0.160	MPHOSPH9
SNOWBALL_006295	-1.35	5.823	0.0018	0.175	EVI5
SNOWBALL_009186	-1.35	1.527	0.0067	0.281	ENSSSCG00000018486
SNOWBALL_007608	-1.35	5.252	0.0015	0.164	SCAF11
SNOWBALL_015469	-1.35	3.434	0.0050	0.257	LOC100515414
SNOWBALL_018712	-1.35	5.186	0.0015	0.164	THG1L
SNOWBALL_018932	-1.35	1.244	0.0035	0.227	ENSSSCG00000020610
SNOWBALL_005344	-1.35	6.072	0.0015	0.163	PSME4
SNOWBALL_011341	-1.35	5.426	0.0013	0.158	ENSSSCG00000008826
SNOWBALL_018125	-1.35	3.556	0.0030	0.215	CWC22
SNOWBALL_032137	-1.35	3.343	0.0054	0.264	ENSSSCG00000024133
SNOWBALL_012761	-1.35	9.679	0.0038	0.236	SC5D
SNOWBALL_047582	-1.34	3.771	0.0053	0.262	KLF3
SNOWBALL_044070	-1.34	1.796	0.0066	0.279	PPM1A
SNOWBALL_007505	-1.34	8.045	0.0020	0.181	NR1H4
SNOWBALL_024058	-1.34	7.113	0.0048	0.255	SLC35F5
SNOWBALL_021596	-1.34	4.616	0.0013	0.159	LOC100739476
SNOWBALL_002569	-1.34	5.070	0.0025	0.200	CDC40
SNOWBALL_035799	-1.34	1.997	0.0043	0.242	
SNOWBALL_012386	-1.34	3.002	0.0047	0.250	ENSSSCG00000029865
SNOWBALL_024477	-1.34	6.141	0.0028	0.210	PPA1
SNOWBALL_002863	-1.34	5.708	0.0066	0.281	ERO1A
SNOWBALL_009013	-1.34	4.318	0.0067	0.281	IMPACT
SNOWBALL_010866	-1.34	6.337	0.0046	0.249	ARHGAP5
SNOWBALL_013239	-1.34	2.283	0.0044	0.244	RGS18
SNOWBALL_043980	-1.34	2.815	0.0065	0.279	
SNOWBALL_018709	-1.34	7.438	0.0033	0.222	ERBB2IP
SNOWBALL_035968	-1.34	4.599	0.0075	0.298	WHSC1L1
mmu-mir-1967	-1.34	2.465	0.0054	0.264	
SNOWBALL_012667	-1.34	5.012	0.0042	0.241	PNPLA8
SNOWBALL_018661	-1.34	5.569	0.0029	0.213	LIFR
SNOWBALL_036078	-1.34	2.014	0.0031	0.218	RICTOR
SNOWBALL_002876	-1.34	7.925	0.0055	0.266	ENSSSCG00000004983
SNOWBALL_035014	-1.34	4.793	0.0048	0.255	PLOD2
SNOWBALL_000364	-1.34	6.166	0.0063	0.279	TLR3
SNOWBALL_022381	-1.34	3.081	0.0054	0.264	LOC106508913
SNOWBALL_000659	-1.34	6.876	0.0021	0.184	IK||WDR55
SNOWBALL_003211	-1.34	5.155	0.0035	0.227	ENSSSCG00000027182
SNOWBALL_018248	-1.34	1.851	0.0051	0.259	
SNOWBALL_022157	-1.34	5.756	0.0047	0.249	BCAP29
SNOWBALL_016639	-1.34	5.440	0.0066	0.279	ADRBK2
SNOWBALL_027043	-1.34	2.774	0.0043	0.244	PCDH11X
SNOWBALL_018827	-1.34	4.962	0.0050	0.257	BDP1

SNOWBALL_039381	-1.34	2.120	0.0020	0.181	
SNOWBALL_021372	-1.34	4.949	0.0059	0.270	ARFGEF2
SNOWBALL_043184	-1.34	2.060	0.0046	0.249	
SNOWBALL_005273	-1.34	1.759	0.0047	0.249	ENSSSCG00000029015
SNOWBALL_010156	-1.34	6.714	0.0062	0.277	IREB2
SNOWBALL_001018	-1.34	5.956	0.0035	0.226	GALNT1
SNOWBALL_001839	-1.34	5.370	0.0023	0.196	ENSSSCG00000012603
SNOWBALL_001806	-1.34	7.167	0.0063	0.279	ATG4A
SNOWBALL_025609	-1.33	4.232	0.0059	0.270	CBL
SNOWBALL_031338	-1.33	4.513	0.0066	0.281	GOSR1
SNOWBALL_028770	-1.33	3.993	0.0046	0.249	LOC106505208
SNOWBALL_009636	-1.33	2.350	0.0027	0.205	
SNOWBALL_044188	-1.33	2.341	0.0039	0.236	
SNOWBALL_036731	-1.33	2.141	0.0040	0.237	
SNOWBALL_011891	-1.33	5.967	0.0043	0.243	KIAA1109
SNOWBALL_004832	-1.33	1.727	0.0029	0.212	LOC100523897
SNOWBALL_031733	-1.33	3.695	0.0076	0.299	LMBRD2
SNOWBALL_024065	-1.33	4.734	0.0019	0.177	ORC4
SNOWBALL_039921	-1.33	5.295	0.0070	0.286	
SNOWBALL_003055	-1.33	5.108	0.0025	0.200	FBXO34
SNOWBALL_047204	-1.33	4.101	0.0034	0.225	
SNOWBALL_013952	-1.33	6.513	0.0059	0.270	FBXL3
SNOWBALL_026256	-1.33	5.066	0.0068	0.282	EIF1AX
SNOWBALL_023004	-1.33	5.780	0.0070	0.287	SMARCA1
SNOWBALL_026634	-1.33	6.142	0.0052	0.260	VGLL4
SNOWBALL_002554	-1.33	5.954	0.0059	0.270	VPS13C
SNOWBALL_029007	-1.33	3.495	0.0066	0.279	CCSAP
SNOWBALL_018051	-1.33	4.735	0.0018	0.175	DOCK10
SNOWBALL_019593	-1.33	6.171	0.0060	0.271	PRKAG2
SNOWBALL_030573	-1.33	3.592	0.0027	0.205	RPL9
SNOWBALL_017109	-1.33	4.191	0.0044	0.244	TAF5
SNOWBALL_032337	-1.32	3.438	0.0038	0.234	RPS6KA3
SNOWBALL_002253	-1.32	6.536	0.0030	0.214	MED23
SNOWBALL_001840	-1.32	4.718	0.0065	0.279	CUL4B
SNOWBALL_002506	-1.32	2.920	0.0029	0.211	TEX9
SNOWBALL_012756	-1.32	3.482	0.0039	0.236	CCDC15
SNOWBALL_005297	-1.32	6.193	0.0047	0.252	
SNOWBALL_047012	-1.32	3.185	0.0068	0.282	
SNOWBALL_006346	-1.32	7.370	0.0035	0.227	MCL1
SNOWBALL_007122	-1.32	6.747	0.0035	0.227	ITPR2
SNOWBALL_033398	-1.32	5.301	0.0067	0.281	
SNOWBALL_012564	-1.32	5.765	0.0054	0.264	
SNOWBALL_002377	-1.32	2.803	0.0069	0.283	HACE1
SNOWBALL_018221	-1.32	6.446	0.0052	0.261	CFLAR
SNOWBALL_035583	-1.32	3.880	0.0073	0.294	LOC102167139
SNOWBALL_033127	-1.32	4.057	0.0057	0.268	
SNOWBALL_024442	-1.32	5.681	0.0038	0.235	AP3M1
SNOWBALL_036226	-1.32	3.609	0.0066	0.279	
SNOWBALL_016122	-1.32	5.480	0.0037	0.233	PCNP
SNOWBALL_013061	-1.32	1.375	0.0025	0.200	ENSSSCG00000026696

SNOWBALL_011593	-1.32	4.626	0.0065	0.279	KLHL2
SNOWBALL_011824	-1.32	7.511	0.0042	0.241	EXOSC9
SNOWBALL_032540	-1.32	3.707	0.0037	0.234	KIF22
SNOWBALL_002443	-1.32	4.902	0.0033	0.222	HACE1
SNOWBALL_012395	-1.32	6.222	0.0059	0.270	RPS25
SNOWBALL_034862	-1.31	4.423	0.0068	0.282	SCOC
SNOWBALL_003215	-1.31	6.316	0.0069	0.284	LOC100154326
SNOWBALL_012740	-1.31	5.176	0.0027	0.205	
SNOWBALL_002497	-1.31	4.710	0.0036	0.227	HDAC2
SNOWBALL_036205	-1.31	1.555	0.0040	0.238	ZC3H12C
SNOWBALL_028374	-1.31	4.701	0.0073	0.294	STRN
SNOWBALL_037817	-1.31	5.144	0.0042	0.242	LOC100622618
SNOWBALL_020656	-1.31	2.928	0.0064	0.279	RAB33A
SNOWBALL_014656	-1.31	6.385	0.0050	0.257	TADA2A
SNOWBALL_024758	-1.31	3.329	0.0039	0.236	TIAL1
SNOWBALL_012749	-1.31	7.864	0.0044	0.244	TMEM243
SNOWBALL_008996	-1.31	3.073	0.0064	0.279	FAM183A
SNOWBALL_019231	-1.31	7.156	0.0033	0.222	RBM39
SNOWBALL_021715	-1.31	5.003	0.0061	0.274	
SNOWBALL_015297	-1.31	3.370	0.0076	0.300	CCDC13
SNOWBALL_022755	-1.31	6.616	0.0055	0.265	ACP5
hsa-mir-1263	-1.31	1.657	0.0057	0.268	
SNOWBALL_030398	-1.31	2.838	0.0076	0.300	STAG2
SNOWBALL_026369	-1.31	6.085	0.0068	0.282	ACAT1
SNOWBALL_043360	-1.31	2.895	0.0071	0.289	LOC100156463
SNOWBALL_010728	-1.31	7.164	0.0068	0.282	PDE8A
SNOWBALL_018748	-1.31	7.628	0.0032	0.219	CNOT8
SNOWBALL_027853	-1.31	4.739	0.0036	0.228	STX17
SNOWBALL_015771	-1.31	5.847	0.0057	0.268	TATDN2
bta-mir-376a	-1.31	0.947	0.0035	0.226	MIR376A
SNOWBALL_043139	-1.31	3.014	0.0042	0.241	
SNOWBALL_002817	-1.31	8.443	0.0051	0.260	CLPX
SNOWBALL_012500	-1.30	5.216	0.0064	0.279	C9H11orf57
SNOWBALL_028828	-1.30	3.558	0.0052	0.261	OGFRL1
SNOWBALL_025241	-1.30	7.692	0.0059	0.270	PNRC1
SNOWBALL_017047	-1.30	3.592	0.0045	0.246	ZNF32
SNOWBALL_023808	-1.30	2.328	0.0042	0.241	ESF1
SNOWBALL_035861	-1.30	5.091	0.0051	0.258	LRPPRC
SNOWBALL_030756	-1.30	3.565	0.0066	0.279	FAIM
SNOWBALL_020960	-1.30	6.039	0.0060	0.273	ATG2A
SNOWBALL_024340	-1.30	2.224	0.0063	0.279	ENSSSCG00000009895
SNOWBALL_045248	-1.30	2.617	0.0043	0.244	
SNOWBALL_011343	-1.30	4.792	0.0058	0.269	ENSSSCG00000008827
SNOWBALL_001846	-1.30	7.843	0.0075	0.298	LAMP2
SNOWBALL_026100	-1.30	6.229	0.0075	0.298	TCP11L1
SNOWBALL_026466	-1.30	7.682	0.0063	0.279	RNF13
SNOWBALL_013034	-1.30	6.551	0.0066	0.279	SRGAP2
SNOWBALL_039297	-1.30	2.139	0.0044	0.244	
SNOWBALL_030434	-1.30	5.701	0.0064	0.279	LOC100622702
SNOWBALL_007151	-1.29	6.335	0.0059	0.270	IPO8

SNOWBALL_009589	-1.29	2.533	0.0072	0.293	SCGN
SNOWBALL_011820	-1.29	5.970	0.0059	0.270	ELF2
SNOWBALL_040870	-1.29	1.926	0.0047	0.251	
SNOWBALL_028197	-1.29	2.143	0.0077	0.300	LOC100737565
SNOWBALL_034180	-1.29	2.701	0.0056	0.266	
SNOWBALL_007165	-1.29	5.154	0.0074	0.295	ENSSSCG00000000529
SNOWBALL_023700	-1.29	6.849	0.0048	0.254	RBM39
SNOWBALL_045659	-1.28	2.611	0.0040	0.237	
SNOWBALL_030028	-1.28	3.584	0.0071	0.289	ENSSSCG00000026382
SNOWBALL_007591	-1.28	5.909	0.0065	0.279	ZDHHC17
SNOWBALL_000094	-1.28	1.930	0.0055	0.266	CD72
bta-mir-181a-1	-1.27	1.340	0.0062	0.278	MIR181A-1
SNOWBALL_003264	-1.27	5.541	0.0073	0.294	LOC106509176
SNOWBALL_003079	-1.27	5.552	0.0063	0.279	KLHL28
SNOWBALL_039070	-1.27	1.562	0.0064	0.279	
SNOWBALL_006767	1.27	5.856	0.0051	0.259	RPS19BP1
SNOWBALL_038831	1.27	5.212	0.0067	0.281	
SNOWBALL_019174	1.27	4.252	0.0074	0.295	MYLK2
SNOWBALL_009376	1.27	7.033	0.0060	0.270	PTPRF
SNOWBALL_027903	1.27	5.009	0.0065	0.279	DPF1
SNOWBALL_034482	1.28	5.562	0.0063	0.279	
SNOWBALL_024084	1.28	5.741	0.0065	0.279	FBRSL1
SNOWBALL_018940	1.28	10.864	0.0062	0.278	ENSSSCG00000020227
SNOWBALL_045880	1.28	1.873	0.0070	0.287	
SNOWBALL_029431	1.28	3.800	0.0072	0.293	LOC100739038
SNOWBALL_005796	1.28	3.450	0.0076	0.300	
SNOWBALL_018837	1.28	3.829	0.0052	0.261	
SNOWBALL_026274	1.28	5.790	0.0055	0.266	
SNOWBALL_039841	1.29	2.346	0.0073	0.294	
SNOWBALL_039516	1.29	2.930	0.0074	0.295	
SNOWBALL_008159	1.29	2.274	0.0070	0.286	PSP-I
SNOWBALL_037117	1.29	2.307	0.0069	0.283	
SNOWBALL_023714	1.29	4.333	0.0052	0.260	GGT7
SNOWBALL_030801	1.29	4.307	0.0056	0.266	ABCB8
SNOWBALL_045403	1.29	2.232	0.0058	0.269	LOC100626202
SNOWBALL_025098	1.29	3.434	0.0066	0.279	SEMA3B
SNOWBALL_008420	1.29	5.317	0.0032	0.219	LIN7B
SNOWBALL_018447	1.29	5.035	0.0048	0.255	C15H2orf57
SNOWBALL_011800	1.29	7.615	0.0057	0.268	
SNOWBALL_028696	1.29	3.741	0.0050	0.257	
SNOWBALL_041363	1.30	2.801	0.0074	0.296	LOC100737722
SNOWBALL_019449	1.30	5.649	0.0063	0.279	ZNF831
SNOWBALL_011619	1.30	2.493	0.0074	0.295	EPHA5
SNOWBALL_026988	1.30	7.390	0.0075	0.296	
SNOWBALL_010738	1.30	5.457	0.0062	0.278	
hsa-mir-3181	1.30	6.332	0.0058	0.269	
SNOWBALL_012799	1.30	6.890	0.0034	0.223	PVRL1
SNOWBALL_040178	1.30	3.650	0.0063	0.279	LOC106505150
SNOWBALL_012537	1.30	8.147	0.0072	0.293	SLC25A13
SNOWBALL_000468	1.30	5.321	0.0050	0.257	ZFP64

SNOWBALL_029370	1.30	5.778	0.0034	0.225	
SNOWBALL_020076	1.30	6.306	0.0065	0.279	ENSSSCG00000012145
SNOWBALL_007870	1.31	6.909	0.0066	0.279	USP10
SNOWBALL_035233	1.31	4.372	0.0072	0.293	
SNOWBALL_000739	1.31	1.830	0.0053	0.263	
SNOWBALL_001063	1.31	5.448	0.0049	0.256	
SNOWBALL_016660	1.31	5.317	0.0045	0.246	ZNRF3
SNOWBALL_031514	1.31	4.340	0.0038	0.234	
SNOWBALL_006715	1.31	5.209	0.0050	0.257	CDPF1
SNOWBALL_037102	1.31	6.676	0.0056	0.266	
SNOWBALL_001047	1.31	3.209	0.0057	0.268	ENSSSCG00000001232
SNOWBALL_024573	1.31	2.069	0.0067	0.281	LYST
SNOWBALL_016416	1.31	6.904	0.0076	0.300	ENSSSCG00000009848
SNOWBALL_023127	1.31	5.557	0.0065	0.279	GZMB
SNOWBALL_005306	1.31	6.701	0.0065	0.279	ENSSSCG00000008485
SNOWBALL_010294	1.31	4.100	0.0067	0.281	TCL1A
SNOWBALL_022349	1.31	2.081	0.0070	0.287	
SNOWBALL_009989	1.31	4.595	0.0054	0.264	SPDEF
SNOWBALL_047313	1.31	4.743	0.0053	0.262	FAM168A
SNOWBALL_025815	1.31	9.063	0.0042	0.241	CDIPT
SNOWBALL_044278	1.31	2.812	0.0053	0.262	ELF4
SNOWBALL_015436	1.31	5.885	0.0034	0.225	CCDC51
SNOWBALL_014188	1.31	8.264	0.0053	0.262	SLC9A3R1
SNOWBALL_041801	1.31	2.844	0.0050	0.258	
SNOWBALL_047546	1.31	6.022	0.0026	0.205	SLC25A37
SNOWBALL_009204	1.32	6.733	0.0041	0.241	MTFR1L
SNOWBALL_030867	1.32	3.318	0.0033	0.222	
SNOWBALL_017831	1.32	8.609	0.0044	0.244	GORASP2
SNOWBALL_045907	1.32	4.162	0.0032	0.220	
SNOWBALL_038494	1.32	2.769	0.0064	0.279	
SNOWBALL_047832	1.32	3.769	0.0061	0.274	EGLN3
SNOWBALL_007827	1.32	4.144	0.0066	0.279	MIRLET7I
SNOWBALL_013358	1.32	6.074	0.0060	0.272	
SNOWBALL_025375	1.32	5.669	0.0055	0.266	
SNOWBALL_001793	1.32	2.278	0.0032	0.220	BEX5
SNOWBALL_001284	1.32	6.370	0.0067	0.281	NELFE
hsa-mir-1208	1.32	3.039	0.0067	0.281	
SNOWBALL_004890	1.32	8.495	0.0057	0.268	CARHSP1
SNOWBALL_012309	1.32	6.716	0.0062	0.277	AQP11
SNOWBALL_010803	1.32	7.072	0.0059	0.270	FOXA1
SNOWBALL_002238	1.32	3.420	0.0015	0.163	GABRR1
SNOWBALL_039035	1.32	8.420	0.0071	0.289	
SNOWBALL_041545	1.32	9.926	0.0075	0.296	
SNOWBALL_016279	1.32	5.628	0.0043	0.244	
SNOWBALL_012322	1.32	3.486	0.0044	0.244	CXCR5
SNOWBALL_023132	1.32	4.788	0.0068	0.282	AGPAT4
SNOWBALL_014821	1.32	5.911	0.0052	0.261	UNC-6||NTN1
SNOWBALL_024709	1.32	2.103	0.0027	0.205	ZC3H12B
SNOWBALL_027447	1.32	4.514	0.0063	0.279	
SNOWBALL_033018	1.33	1.704	0.0036	0.227	ESRRG

hsa-mir-133a-2	1.33	1.278	0.0039	0.236	
bta-mir-3603	1.33	2.148	0.0022	0.192	
SNOWBALL_023939	1.33	8.549	0.0046	0.249	GORASP2
SNOWBALL_025684	1.33	5.834	0.0023	0.197	
SNOWBALL_037591	1.33	4.383	0.0027	0.205	
hsa-mir-23b	1.33	6.354	0.0030	0.215	
SNOWBALL_023668	1.33	3.940	0.0039	0.236	L3MBTL1
SNOWBALL_027893	1.33	3.609	0.0052	0.261	
SNOWBALL_046527	1.33	4.354	0.0025	0.200	
SNOWBALL_035302	1.33	7.403	0.0076	0.300	
SNOWBALL_016872	1.33	3.753	0.0059	0.270	KCNIP2
SNOWBALL_001011	1.33	3.073	0.0033	0.222	PDPN
SNOWBALL_040940	1.33	5.542	0.0024	0.200	
SNOWBALL_047032	1.33	2.182	0.0039	0.236	
SNOWBALL_039387	1.33	3.384	0.0031	0.217	
SNOWBALL_017918	1.33	4.876	0.0046	0.248	METAP1D
SNOWBALL_016564	1.33	5.972	0.0025	0.200	TRMT2A
SNOWBALL_004792	1.33	7.731	0.0019	0.179	SLC9A3R2
SNOWBALL_011118	1.33	5.274	0.0035	0.227	ZFYVE21
SNOWBALL_005536	1.33	5.198	0.0041	0.241	ENSSSCG00000005895
SNOWBALL_037075	1.34	3.734	0.0070	0.287	
SNOWBALL_034834	1.34	5.772	0.0049	0.256	CABLES1
SNOWBALL_029545	1.34	5.475	0.0044	0.244	MFNG
SNOWBALL_047555	1.34	2.381	0.0020	0.181	LOC102163478
SNOWBALL_020913	1.34	6.484	0.0064	0.279	
SNOWBALL_029117	1.34	3.736	0.0032	0.220	OLFM1
SNOWBALL_005087	1.34	6.731	0.0025	0.200	
SNOWBALL_019808	1.34	5.445	0.0050	0.257	ARF5
SNOWBALL_016721	1.34	5.106	0.0031	0.217	ARVCF
SNOWBALL_020456	1.34	1.488	0.0044	0.244	ENSSSCG00000020209
SNOWBALL_023619	1.34	5.738	0.0033	0.222	
SNOWBALL_030786	1.34	4.045	0.0065	0.279	
SNOWBALL_033327	1.34	4.417	0.0041	0.241	NYNRIN
SNOWBALL_038028	1.34	4.667	0.0043	0.244	NYNRIN
bta-mir-1281	1.34	6.221	0.0058	0.269	
SNOWBALL_013449	1.34	6.309	0.0062	0.278	MOB3B
SNOWBALL_013175	1.34	1.652	0.0042	0.241	APOBEC4
SNOWBALL_026003	1.34	4.948	0.0030	0.215	C13H21orf2
bta-mir-2432	1.34	1.919	0.0039	0.236	
SNOWBALL_004249	1.34	7.263	0.0036	0.227	GET4
SNOWBALL_017105	1.34	3.165	0.0058	0.269	ENSSSCG00000023865
SNOWBALL_000074	1.35	3.684	0.0027	0.205	
SNOWBALL_016442	1.35	6.095	0.0011	0.141	SH2B3
SNOWBALL_012746	1.35	9.513	0.0048	0.255	OAF
SNOWBALL_022743	1.35	4.443	0.0057	0.268	MYO9A
SNOWBALL_037755	1.35	3.679	0.0055	0.266	RNF130
SNOWBALL_026570	1.35	5.525	0.0022	0.189	
SNOWBALL_002465	1.35	5.463	0.0030	0.215	RBPMS2
SNOWBALL_025627	1.35	7.099	0.0045	0.246	B4GALT1
SNOWBALL_025622	1.35	8.305	0.0029	0.212	

SNOWBALL_003288	1.35	5.597	0.0014	0.162	TTC39B
SNOWBALL_022318	1.35	6.393	0.0034	0.225	
SNOWBALL_001155	1.35	6.388	0.0024	0.200	
SNOWBALL_030590	1.35	2.888	0.0046	0.249	
SNOWBALL_023021	1.35	5.055	0.0074	0.294	DDIT3
SNOWBALL_037573	1.35	2.670	0.0025	0.200	
SNOWBALL_034101	1.36	4.943	0.0009	0.128	MYO1C
SNOWBALL_019224	1.36	5.484	0.0015	0.165	GNRH2
SNOWBALL_024283	1.36	5.370	0.0015	0.163	KREMEN1
SNOWBALL_017499	1.36	2.034	0.0049	0.257	ENSSSCG00000019987
SNOWBALL_038077	1.36	2.116	0.0025	0.200	
hsa-mir-654	1.36	1.977	0.0068	0.282	
SNOWBALL_041677	1.36	2.858	0.0043	0.244	
SNOWBALL_002121	1.36	5.208	0.0032	0.219	ESR1
hsa-mir-1298	1.36	1.381	0.0024	0.199	
SNOWBALL_016106	1.36	6.074	0.0011	0.144	MX1
SNOWBALL_010274	1.36	7.183	0.0028	0.210	ACOT6
SNOWBALL_024667	1.36	8.147	0.0052	0.260	
SNOWBALL_017386	1.36	1.440	0.0064	0.279	PCGF5
SNOWBALL_014350	1.36	5.296	0.0068	0.282	FTSJ3
SNOWBALL_003697	1.36	5.305	0.0028	0.207	ARPC5L
SNOWBALL_005580	1.37	8.120	0.0031	0.217	EXOSC4
bta-mir-411	1.37	1.951	0.0055	0.266	
SNOWBALL_019709	1.37	7.410	0.0035	0.227	CREB3L2
SNOWBALL_032970	1.37	5.230	0.0056	0.266	
SNOWBALL_044891	1.37	2.786	0.0024	0.198	GRAMD1B
bta-mir-2344	1.37	3.225	0.0067	0.281	
SNOWBALL_039480	1.37	7.160	0.0044	0.244	SLC26A1
SNOWBALL_007513	1.37	1.881	0.0052	0.261	ENSSSCG00000020533
SNOWBALL_025056	1.37	4.410	0.0073	0.294	PRRT3
SNOWBALL_028751	1.37	5.791	0.0033	0.222	TRAPPC12
SNOWBALL_024528	1.37	1.861	0.0024	0.197	ZNF37A
SNOWBALL_005443	1.37	6.665	0.0013	0.159	SDC1
hsa-mir-665	1.37	3.878	0.0047	0.249	
SNOWBALL_032491	1.37	3.520	0.0007	0.115	
SNOWBALL_025614	1.37	7.945	0.0029	0.213	STEAP3
SNOWBALL_004828	1.37	7.396	0.0017	0.173	FAM195A
SNOWBALL_009466	1.37	6.415	0.0016	0.167	SLC22A23
SNOWBALL_020457	1.37	4.937	0.0012	0.149	MXRA5
bta-mir-885	1.37	5.686	0.0059	0.270	MIR885
SNOWBALL_026382	1.37	3.038	0.0042	0.241	
SNOWBALL_015247	1.38	4.029	0.0035	0.226	FAM198A
bta-mir-92b	1.38	6.502	0.0020	0.182	MIR92B
mmu-mir-802	1.38	1.362	0.0042	0.241	
SNOWBALL_001681	1.38	3.416	0.0012	0.147	ENSSSCG00000031040
bta-mir-219-1	1.38	4.552	0.0040	0.240	MIR219B
SNOWBALL_004236	1.38	7.450	0.0043	0.244	C3H7orf50
SNOWBALL_016177	1.38	5.659	0.0014	0.161	MIR885
SNOWBALL_016699	1.38	9.324	0.0033	0.222	SEC14L2
SNOWBALL_044843	1.38	6.343	0.0027	0.205	

SNOWBALL_012143	1.38	4.330	0.0036	0.229	ENSSSCG00000014790
SNOWBALL_019938	1.38	1.404	0.0043	0.244	ENSSSCG00000020206
SNOWBALL_032042	1.38	1.819	0.0013	0.154	
SNOWBALL_027228	1.39	6.674	0.0012	0.149	
SNOWBALL_031401	1.39	3.061	0.0005	0.101	
SNOWBALL_015105	1.39	5.929	0.0045	0.246	UNC-6
SNOWBALL_002404	1.39	6.021	0.0022	0.191	RWDD2A
SNOWBALL_017696	1.39	1.611	0.0064	0.279	DLGAP2
SNOWBALL_025273	1.39	6.965	0.0040	0.237	ENSSSCG00000022404
SNOWBALL_024059	1.39	5.827	0.0042	0.241	GPR39
SNOWBALL_004327	1.39	2.592	0.0044	0.244	LOC102164877
SNOWBALL_000932	1.39	3.015	0.0033	0.222	LOC100511326
SNOWBALL_008290	1.39	4.157	0.0027	0.205	LENG9
SNOWBALL_047704	1.39	6.723	0.0054	0.263	RBM47
SNOWBALL_016808	1.39	4.092	0.0009	0.124	FAM222A
SNOWBALL_043777	1.39	4.153	0.0008	0.121	
SNOWBALL_040633	1.39	3.360	0.0006	0.111	
bta-mir-2371	1.39	1.367	0.0019	0.177	
SNOWBALL_005131	1.39	3.219	0.0016	0.166	VIT
SNOWBALL_021527	1.39	5.158	0.0027	0.205	ENSSSCG00000025663
SNOWBALL_015712	1.40	2.246	0.0038	0.236	IL5RA
bta-mir-200b	1.40	2.134	0.0031	0.218	ENSSSCG00000024128
SNOWBALL_000083	1.40	2.516	0.0055	0.266	
SNOWBALL_007634	1.40	4.683	0.0035	0.226	ENSSSCG00000000747
SNOWBALL_003243	1.40	6.499	0.0013	0.154	PARP16
SNOWBALL_015843	1.40	7.106	0.0007	0.117	B3GALT5
SNOWBALL_045333	1.40	3.181	0.0009	0.131	
SNOWBALL_040967	1.40	3.594	0.0006	0.114	
SNOWBALL_015768	1.41	3.525	0.0031	0.217	CLSTN2
SNOWBALL_013502	1.41	7.788	0.0019	0.179	B4GALT1
SNOWBALL_025936	1.41	5.985	0.0005	0.101	ILK
SNOWBALL_044790	1.41	7.510	0.0036	0.229	SYVN1
SNOWBALL_041258	1.41	2.480	0.0020	0.181	
SNOWBALL_026567	1.41	4.006	0.0005	0.101	
SNOWBALL_021137	1.41	9.197	0.0040	0.237	TDH
SNOWBALL_031732	1.41	6.003	0.0005	0.104	EXD3
SNOWBALL_037183	1.41	4.601	0.0013	0.153	
bta-mir-2376	1.41	1.636	0.0006	0.111	
SNOWBALL_010014	1.41	7.216	0.0027	0.205	POLH
SNOWBALL_026039	1.41	6.289	0.0015	0.165	MRPL45
SNOWBALL_025031	1.41	5.871	0.0004	0.096	EXTL3
SNOWBALL_008816	1.41	4.047	0.0011	0.144	CABYR
SNOWBALL_023167	1.42	3.183	0.0008	0.121	CD1E
SNOWBALL_014298	1.42	5.601	0.0014	0.160	DCAKD
SNOWBALL_031164	1.42	2.479	0.0018	0.175	LOC100625164
SNOWBALL_035016	1.42	2.629	0.0005	0.100	
SNOWBALL_004857	1.42	7.615	0.0021	0.182	ANKS3
SNOWBALL_010122	1.42	1.678	0.0006	0.112	CRISP1
SNOWBALL_015926	1.42	3.477	0.0014	0.160	FAM3B
SNOWBALL_045426	1.42	5.939	0.0002	0.065	PER3

SNOWBALL_020051	1.42	3.599	0.0015	0.164	TCEANC
SNOWBALL_011390	1.42	7.263	0.0021	0.184	RBM47
bta-mir-453	1.43	4.772	0.0025	0.200	
SNOWBALL_003840	1.43	3.518	0.0014	0.160	PRDM12
SNOWBALL_017677	1.43	7.413	0.0015	0.162	GPR39
SNOWBALL_019363	1.43	2.575	0.0019	0.179	ENSSSCG00000018845
SNOWBALL_005213	1.43	7.681	0.0004	0.089	
SNOWBALL_012231	1.43	6.274	0.0003	0.086	LOC100738084
SNOWBALL_015229	1.43	3.651	0.0006	0.109	ENSSSCG00000011268
SNOWBALL_035824	1.44	3.433	0.0004	0.090	
mmu-mir-701	1.44	1.454	0.0008	0.120	
SNOWBALL_018767	1.44	2.904	0.0013	0.156	GABRA1
SNOWBALL_047512	1.45	3.972	0.0007	0.115	
SNOWBALL_014707	1.45	4.652	0.0010	0.138	SGCA
SNOWBALL_023421	1.46	6.173	0.0017	0.173	MMP7
SNOWBALL_024285	1.46	6.232	0.0004	0.099	ZNRF3
SNOWBALL_026827	1.47	4.048	0.0002	0.075	
SNOWBALL_012032	1.47	5.158	0.0002	0.068	ARFIP2
SNOWBALL_023400	1.47	5.134	0.0006	0.115	BYSL
SNOWBALL_014952	1.47	5.256	0.0007	0.117	COX10
bta-mir-665	1.47	5.362	0.0009	0.124	
SNOWBALL_014788	1.47	1.603	0.0002	0.067	ENSSSCG00000018170
SNOWBALL_010901	1.48	4.975	0.0004	0.099	SLC41A3
SNOWBALL_015928	1.48	1.540	0.0003	0.083	ENSSSCG00000019913
hsa-mir-220c	1.48	5.415	0.0003	0.086	
SNOWBALL_006183	1.48	1.332	0.0002	0.072	ENSSSCG00000019445
SNOWBALL_026619	1.48	3.296	0.0018	0.176	
SNOWBALL_000975	1.49	7.087	0.0001	0.054	DNAJA2
SNOWBALL_031400	1.49	5.139	0.0002	0.065	TNFRSF19
SNOWBALL_021042	1.50	5.072	0.0013	0.154	SLC7A9
SNOWBALL_014647	1.50	3.896	0.0002	0.069	GSDMA
SNOWBALL_013129	1.50	4.525	0.0011	0.144	LOC102166466
SNOWBALL_025338	1.50	6.242	0.0009	0.124	
bta-mir-2383	1.50	5.548	0.0004	0.089	
SNOWBALL_010582	1.50	5.744	0.0006	0.115	EGLN3
SNOWBALL_024870	1.50	3.082	9.33E-05	0.045	KCNIP2
hsa-mir-622	1.52	5.472	0.0004	0.096	
hsa-mir-4252	1.52	4.473	0.0003	0.087	
SNOWBALL_000160	1.54	8.199	6.44E-05	0.042	PDK2
SNOWBALL_009881	1.56	5.526	0.0001	0.056	KCNK5
mmu-mir-1193	1.56	3.934	9.28E-05	0.045	
hsa-mir-4304	1.58	4.876	0.0002	0.069	
SNOWBALL_013601	1.60	2.581	0.0002	0.068	ENSSSCG00000020151
SNOWBALL_006697	1.61	4.312	3.13E-05	0.030	CELSR1
SNOWBALL_006991	1.61	6.813	2.53E-05	0.028	ITGA5
mmu-mir-1964	1.62	4.385	9.88E-05	0.046	
SNOWBALL_005621	1.63	5.038	1.73E-05	0.024	SYBU
SNOWBALL_030212	1.63	7.234	8.93E-05	0.045	
SNOWBALL_004677	1.63	6.253	1.93E-05	0.024	TBL3
SNOWBALL_022900	1.65	6.134	1.17E-05	0.020	CLDN3

SNOWBALL_022577	1.65	4.088	5.46E-05	0.039	LOC106508719
SNOWBALL_000565	1.65	5.249	8.20E-05	0.044	GNAS
hsa-mir-219-1	1.67	2.929	4.89E-06	0.014	
SNOWBALL_000187	1.75	5.920	5.56E-06	0.014	RNF43
SNOWBALL_026023	1.76	3.267	2.30E-05	0.027	
SNOWBALL_023059	1.84	2.343	5.86E-06	0.014	
SNOWBALL_026778	1.87	5.411	1.76E-06	0.010	SLC7A9

Supplementary Table 2. Composition and chemical analysis of the diet used in the study

**Diet composition (g/kg)**	
Barley	385.4
Wheat	404.0
Soya	175.0
Soya oil	10.0
Lysine HCl (78.8)	4.0
DL-Methionine	1.0
L-Threonine (98)	1.5
L-Tryptophan	0.0
Vitamin and mineral mix[1]	1.0
Natuphos 5000 FTU/g[2]	0.1
Salt feed grade	3.0
Dicalcium phosphate	2.0
Limestone flour	13.0
**Chemical analysis (g/kg dry matter)**	
Crude protein	205.4
Crude fibre	37.5
Crude ash	45.4
Ether extract	27.2
Digestible energy (MJ/kg)[3]	16.0
Net energy (MJ/kg)[3]	9.8
**Amino acids (g/kg)**	
Lysine	11.1
Methionine	3.6
Methionine + cysteine	6.8
Threonine	7.5
Tryptophan	2.2

[1]Premix provided per kg of complete diet: Cu, 15 mg; Fe, 24 mg; Mn, 31 mg; Zn, 80 mg, I, 0.3 mg; Se, 0.2 mg; vitamin A, 2000 IU; vitamin D_3, 500 IU; vitamin E, 40 IU; vitamin K, 4 mg; vitamin B_{12}, 15 µg; riboflavin, 2 mg; nicotinic acid, 12 mg; pantothenic acid, 10 mg; vitamin B_1, 2 mg; vitamin B_6, 3 mg.

[2]Phytase; 5000 FTU/g equal to 500 FTU per kg finished feed.

[3]Digestible energy and net energy were calculated from book values.

Diets were pelleted to 3mm diameter after steam conditioning to 50-55 °C.

Supplementary Table S3. Primers used in RT-qPCR to verify microarray results

Primer	Sequence	Annealing temperature	Amplicon length	Fragment type
Ssc_ITGA5_f	GGAGGCAGAATGTTTCAGGC	63°C	168	qRT-PCR
Ssc_ITGA5_r	TAGGGCATCTTCAGGGCTTG			
Ssc_ITGA5_f	AGGGTCACAGGACTCAGCAAC	63°C	444	Standard
Ssc_ITGA5_r	CACGAGGATGGCTAGGATAATG			
Ssc_NR1H4_	TGACTCAAGAAGAATACGCTCTGC	60°C	120	qRT-PCR
Ssc_NR1H4_	TTTGTAGCACCTCAAGCAGTGG			
Ssc_NR1H4_	CGAAAGAGTGGTATCTCCGATG	60°C	339	Standard
Ssc_NR1H4_	GAGAAGCGGGGTAAACTTGTG			
Ssc_SLC1A4_	TCTCTTTGATCCTGGCTGTGG	60°C	107	qRT-PCR
Ssc_SLC1A4_	CTTCTGGTTCAGGTGGTGGAG			
Ssc_SLC1A4_	CTCACCATCGCCATTATCCTG	60°C	573	Standard
Ssc_SLC1A4_	CTGGAGGGCGTGATTACAAAC			
Ssc_SLC7A9_	CGCTATAAGTTCGGATGGGC	60°C	108	qRT-PCR
Ssc_SLC7A9_	CTGAGCTTGTTACTCTGGCGG			
Ssc_SLC7A9_	CCCCTGCCATCATCTTTCAC	60°C	489	Standard
Ssc_SLC7A9_	AGGAAGAAATAGCCACAAATACTGC			
Ssc_SQLE_f1	TGTGAATGTCCTTGCTCAGG	60°C	196	qRT-PCR
Ssc_SQLE_r1	GGCATAGACTGCAACAGCAA			
Ssc_SQLE_f2	GGGCATCCCTGACCTTTATG	60°C	565	Standard
Ssc_SQLE_r2	TTCAAAAACAAATCAATCCCAATC			
Ssc_RPL10_f	CTGTGTTCGTCTTTTCTTCC	60°C	199	qRT-PCR
Ssc_RPL10_r	TCATCCACTTTTGCCTTCT			
Ssc_RPL32_f	AGCCCAAGATCGTCAAAAAG	60°C	165	qRT-PCR
Ssc_RPL32_r	TGTTGCTCCCATAACCAATG			

ITGA5 – Integrin, Alpha 5; *NR1H4* – Nuclear Receptor Subfamily 1 Group H Member 4; *SLC1A4* – Solute Carrier Family 1 Member 4; *SLC7A9* – Solute Carrier Family 7 Member 9; *SQLE* – Squalene Epoxidase; *RPL10* – Ribosomal protein 10; *RPL32* - Ribosomal protein 32
